# Supplementary material for: New Insights into Immunological Involvement in Congenital Disorders of Glycosylation (CDG) from a People-Centric Approach
Source: J Clin Med. 2020 Jul 3;9(7):2092. doi: 10.3390/jcm9072092 (PMC7408855; doi:10.3390/jcm9072092)
Supplement: Supplementary file 1 [file jcm-09-02092-s001.pdf]

New insights into immunological involvement in Congenital Disorders of Glycosylation (CDG) from a people-centric approach

Supplementary Material

Index of tables

Table S1 – **Questionnaire dissemination and recruitment** 1

Table S2 – **Annotation of *PMM2* variants in databases** 2

Table S3 – **Mutation pathogenicity prediction *in silico* tools** 4

Table S4 – **Characterization of deceased PMM2-CDG patients** 6

Table S5 – **PMM2-CDG patients with confirmed autoimmune manifestations** 7

Table S6 – **Immune-related manifestations and other clinical data in less frequent CDG (non-PMM2-CDG group)** 8

Table S7 – **Characteristics of the control, PMM2-CDG and non-PMM2-CDG groups with relevant infections** 16

Table S8 – **Characteristics of the control, PMM2-CDG and non-PMM2-CDG groups with allergies** 16

Table S9 – **Correlation of PMM2-CDG clinical features with infections and allergies in PMM2-CDG** 17

Table S10 - **Correlation of clinical features with overall phenotypic severity in PMM2-CDG** 18

Table S11 – **Immune-related manifestations and other clinical data of PMM2-CDG patients harboring the Arg141His/R141H variant** 19

Table S12 - **Immune-related manifestations and other clinical data of PMM2-CDG patients not harboring the Arg141His/R141H variant.** 23

Table S13 – **Analysis of the pathogenicity of *PMM2* variants listed in the ImmunoCDGQ with a set of *in silico* prediction tools.** 27

Table S14 – **Comparative analysis of the effects of wild-type vs mutated residues on PMM2 physico-chemical and structural properties and overall predicted impact on the function/activity of the protein.** 30

Table S15 - **Laboratory alterations found among control and PMM2-CDG participants** 34

Table S16 – **Information needs related to immunology topics among control, PMM2-CDG and non-PMM2-CDG participants according to immune status** 35

New insights into immunological involvement in Congenital Disorders of Glycosylation (CDG) from a people-centric approach
Supplementary Material

Index of figures

Figure S1 – **Characteristics of the ImmunoCDGQ (PMM2-CDG and non-PMM2-CDG groups) and ImmunoHealthyQ (control group) participants.** 36

Figure S2 - **Dietary supplementation impact on immunological dysfunction in PMM2-CDG and non-PMM2-CDG patients.** 37

Figure S3 - **Infection profile (frequency and severity classification).** 38

Figure S4 – **Organ-specific infections reported by control and PMM2-CDG at the time of participation in the study** 39

Figure S5 – **Age impact on infection and allergy prevalence and severity.** A) Infection modulation by age; B) Allergy modulation by age. While for infections a tendential positive impact of ageing on infection severity/frequency for allergy that tendency is not as marked, both for PMM2-CDG and controls. 40

Figure S6 – **Infection types (per organ/system) at the time when infections were more frequent/severe and infection-caused permanent damage.** 41

Figure S7 – **Time to cure infections, their association with seasons and causing infectious agents.** 42

Figure S8 – **Infection treatments and actions taken upon infection suspicion**. 43

Figure S9 - **Allergy prevalence.** 44

Figure S11 - **Vaccination adherence and monitoring**. 46

Figure S12 – **Immune awareness**. 47

Figure S13 - **Immune testing**. 48

Figure S14 – **Immunology-related information needs, glossary use, questionnaire understandability and management.** 49

Figure S15 – **Overall analysis of respondents' views about the questionnaires' understandability, glossary use/usefulness, and their participation experience/management.** 50

Table S1 – **Questionnaire dissemination and recruitment.** The channels used and institutions involved in the dissemination and recruitment campaigns of the ImmunoCDG and ImmunoHealthyQ.

Information and recruitment campaign	**ImmunoCDGQ**	
**Webpage**	https://www.researchcdg.com/immunocdgq.html	http
**Social media channels**	**Facebook:** Síndrome CDG page and "CDG Global Alliance" closed Facebook group<br>**Twitter:** @worldCDG<br>**LinkedIn:** Portuguese Association CDG<br>**RareConnect:** CDG Community	**Fa**<br>ap
**Emailing**	National CDG patient associations	Vi
**Additional strategies/media**	Teleconferences with clinicians and reference CDG patient associations	In

**Institutions who actively participated in the dissemination**	National CDG patient associations, CDG & Allies-Professionals and Patient Associations International Network (CDG & Allies -PPAIN), European reference network for rare metabolic diseases (MetabERN)	Cl

Table S2 – **Annotation of *PMM2* variants in databases.** *PMM2* variants have a good database coverage. Two novel mutations in one patient were identified. Search in all databases was done from August 2019 to February 2020.

**Mutation**		**Nº of patients (ImmunoCDGQ)**	**Databases**		
**Transcript Variant**	**Protein Variant**		**HGMD Annotation**	**LOVD3 Annotation**	**ClinVar Annotation**
c.422G>A[α]	Arg141His/R141H	40	DM	Pathogenic	Pathogenic
c.341G>A	Arg114His/R114H	1	DM	NR	Pathogenic
c.338C>T	Pro113Leu/P113L	11	DM	Pathogenic	Pathogenic
c.691G>A	Val231Met/V231M	11	DM	Pathogenic	Pathogenic
c.323C>T	Ala108Val/A108V	6	DM	Pathogenic	Pathogenic/likely pathogenic
c.368G>A	Arg123Gln/R123Q	6	DM	Pathogenic	*Conflicting interpretations of pathogenicity* Pathogenic (5); Likely pathogenic (1); VUS (1)
c.710C>T	Thr237Met/T237M	5	DM	Pathogenic	Pathogenic/Likely pathogenic
c.470T>C	Phe157Ser/F157S	4	DM	Pathogenic	Pathogenic/Likely pathogenic
c.357C>A[β]	Phe119Leu/F119L	4	DM	Pathogenic	Pathogenic

Mutation		Nº of patients (ImmunoCDGQ)	Databases		
Transcript Variant	Protein Variant		HGMD Annotation	LOVD3 Annotation	ClinVar Annotation
c.205C>T	Pro69Ser/P69S	3	DM	Pathogenic	*Conflicting interpretations of pathogenicity* Likely pathogenic (1); VUS (1)
c.710C>G[γ]	Thr237Arg/T237R	3	DM	Pathogenic/Likely pathogenic	Pathogenic
c.415G>A[†]	Glu139Lys/E139K	3	DM	NR	Pathogenic/Likely pathogenic
c.395T>C	Ile132Thr/I132T	2	DM	Pathogenic	Likely pathogenic
c.647A>T	Asn216Ile/N216I	2	DM	Pathogenic	Pathogenic
c.640G>A	Gly214Ser/G214S	2	DM	Pathogenic/VUS	VUS
c.548T>C	Phe183Ser/F183S	2	DM	Pathogenic	Pathogenic
c.385G>A	Val129Met/V129M	2	DM	Pathogenic	Pathogenic/likely pathogenic
c.677C>G	Thr226Ser/T226S	2	DM	Pathogenic	Pathogenic
c.620T>C	Phe207Ser/F207S	2	DM	NR	Likely pathogenic
c.95T>G	Leu32Arg/L32R	2	DM	NR	Pathogenic
c.640-9T>G	IVS7-9T	2	DM	NR	Pathogenic
c.442G>A	Asp148Asn/D148N	1	DM	Pathogenic	Pathogenic/likely pathogenic
c.563A>G[μ]	Asp188Gly/D188G	1	DM	Pathogenic	Pathogenic/likely pathogenic
c.227A>G	Tyr76Cys/Y76C	1	DM	NR	NR
c.430T>C	Phe144Leu/F144L	1	DM	Pathogenic	Likely pathogenic
c.140C>T[#]	Ser47Leu/S47L	1	NR	NR	NR
c.98A>C[#]	Gln33Pro/Q33P	1	NR	NR	NR
c.127G>A	Val43Met/V43M	1	DM	NR	Likely pathogenic
c.623G>C	Gly208Ala/G208A	1	DM	Pathogenic	Pathogenic/likely pathogenic
c.367C>T	Arg123Ter/R123*	1	DM	Pathogenic	Pathogenic
c.193G>T	Asp65Tyr/D65Y	1	DM	Pathogenic	Pathogenic
c.191A>G	Tyr64Cys/Y64C	1	DM	NR	NR

Mutation		Nº of patients (ImmunoCDGQ)	Databases		
Transcript Variant	Protein Variant		HGMD Annotation	LOVD[3] Annotation	ClinVar Annotation
c.484C>T	Arg162Trp/R162W	1	DM	Pathogenic	Pathogenic/likely pathogenic
c.667G>A	Asp223Asn/D223N	1	DM	Pathogenic	NR
c.26G>A	Cys9Tyr/C9Y	1	DM	Pathogenic	Pathogenic/likely pathogenic
c.61C>G	Arg21Gly/R21G	1	DM	Pathogenic	VUS
c.511dupA	Thr171AsnfsTer11/ T171Nfs*11	1	NR	NR	Pathogenic/likely pathogenic
Total		66	34	26	31

Legend: DM – Damaging; NR – Not reported; VUS – Variant of uncertain significance

[×] Results displayed here derive from the ACMG Classification available on the VarSome platform. ClinVar (with a rating of at least 1 star) and UniProt options were activated in the ACMG Classification.

[α] Described in Expasy as a frequent mutation causing loss of activity. Only observed in heterozygosity, being homozygosity incompatible with life.

[β] Described in Expasy as resulting in partial loss of activity.

[γ] Described in Expasy as leading to loss of activity.

[†] Described in Expasy as causing the disruption of a splicing enhancer sequence, hence mostly resulting in a protein with exon 5 skipped with a slight activity reduction.

[μ] Described in Expasy as being severe.

[≠] Novel variants – These variants had never been annotated neither in any of the databases searched nor in the literature.

Table S3 – **Mutation pathogenicity prediction *in silico* tools.** Multiple pathogenicity prediction tools – using distinct methods and biological data sources/parameters - have been employed to i) give a broader biological perspective and ii) overcome the limitations of each individual tool. This strategy allows for a more accurate differentiation between neutral and damaging variants as well as acts as a safeguard mechanism to prevent false positive and negative results.

Tool	Method	Score Qualitative
**Single nucleotide variants (SNVs) and single nucleotide polymorphisms (SNPs)**		
Deleterious annotation of genetic variants using neural networks (DANN)$^{\alpha}$	Uses deep neural networks to recognize both coding and non-coding pathogenic variants based on genomic data (e.g. genome wide association studies), capturing non-linear relationships among the features.	NA
Functional Analysis through Hidden Markov Models (FATHMM)-MKL$^{\alpha}$	Employs multiple kernel learning to map conservation, epigenomic signals, disease-associated and functionally neutral amino acids mapping onto conserved protein domains. MKL also predicts noncoding effects by	i) Damaging and ii) Neutral

	integrating functional annotation information from the Encyclopaedia of DNA Elements (ENCODE).	
Have (y)Our Protein Explained (HOPE) https://www3.cmbi.umcn.nl	Predicts the structural effects of a mutation by retrieving information from several webservers and databases (e.g. UniProt and Expasy)	Gives a qualitative prediction mainly basec conservation and amino acid similarities
Mutation Taster[µ] http://www.mutationtaster.org/	This Naïve Bayes classifier-based tool incorporates evolutionary conservation, splice-site, mRNA, protein and regulatory features to predict variant pathogenicity	i) Disease causing; ii) Disease causing autom iii) Polymorphism and iv) Polymorphism automatic
Protein Variation Effect Analyzer (PROVEAN) http://provean.jcvi.org/	Bases its predictions on sequence homology using delta alignment score	i) Deleterious and ii) Tolerated
Polyphen-2 (HumVar)* http://genetics.bwh.harvard.edu/pph2/	Relies on Bayesian methods to predict the possible impact of an amino acid substitution on the structure and function of a human protein using straightforward physical and comparative considerations. The HumVar score encompasses 13,032 human disease-causing mutations from UniProt and 8,946 human nonsynonymous SNPs.	i) Probably damaging, ii) Possibl damaging and iii) Benign
Sorting Intolerant from Tolerant (SIFT) https://sift.bii.a-star.edu.sg/	Utilizes position specific scoring matrixes to identify nonsynonymous variants based on sequence homology (i.e. sequence conservation) derived from closely related sequences collected through PSI-BLAST.	i) Damaging and ii) Tolerated
**Insertions/deletions (INDELs)**		
SIFT Indels https://sift.bii.a-star.edu.sg/	Uses a decision tree algorithm to distinguish between disease-causing and neutral coding indels. Currently, this tool can make predictions for both frameshifting and in-frame indels. Built on UCSC pairwise alignments of the human genome with mammalian genomes.	i) Damaging and ii) Neutral
Variant Effect Scoring (VEST 4) – Indel http://www.cravat.us/CRAVAT/	A random forest algorithm was trained to predict pathogenicity of in-frame and frameshift indels, considering measures of gene importance, the damaging effect of the variant on protein activity, evolutionary conservation and protein local environment.	i) Pathogenic and ii) Benign
**Splicing mutations**		

Human Splicing Finder (HSF) http://www.umd.be/HSF/	Employs a set of 12 algorithms (e.g. RESCUE-ESSE and ESE-Finder) to predict the effects of mutations on splicing signals/motifs or to identify splicing motifs in any human sequence. Capable of analyzing the effects of SNPs on splicing.	Gives information of the type of signal dete namely: i) Donor or acceptor splice site; i Branch point site; iii) Exonic Splicing Enhan (ESE) and iv) Exonic Splicing Silencers (E

Legend: INDELs – Insertions/deletions; NA – Not applicable/available; SNPs – Single nucleotide polymorphisms; SNVs – Single nucleotide variants

$^{\alpha}$Obtained directly at the VarSome platform (https://varsome.com)

$^{\mu}$Mutation Taster was also used to predict INDEL pathogenicity since this tool also allows for INDEL analysis

*The HumVar score was used in this study because it was necessary to differentiate mutations with drastic effects from other human variations, including abundant mildly deleterious alleles involved in Mendelian disorders.

$^{\#}$Authors did not specify a threshold for an Indel to be considered damaging. However, they did state that INDEL pathogenicity can be more challenging to predict and that regarding frameshift INDELs particularly, common INDELs are gene-damaging and an allele frequency threshold of 0.05 may be too low.

$^{\beta}$Detailed information available at http://www.umd.be/HSF3/technicaltips.html

References

1. Quang, D.; Chen, Y.; Xie, X. DANN: A deep learning approach for annotating the pathogenicity of genetic variants. *Bioinformatics* **2015**, *31*, 761–763. doi:10.1093/bioinformatics/btu703.
2. Sun, H.; Yu, G. New insights into the pathogenicity of non-synonymous variants through multi-level analysis. *Sci. Rep.* **2019**, *9*. doi:10.1038/s41598-018-38189-9.
3. Shihab, H.A.; Rogers, M.F.; Gough, J.; Mort, M.; Cooper, D.N.; Day, I.N.M.; Gaunt, T.R.; Campbell, C. An integrative approach to predicting the functional effects of non-coding and coding sequence variation. *Bioinformatics* **2015**, *31*, 1536–1543. doi:10.1093/bioinformatics/btv009.
4. Liu, X.; Wu, C.; Li, C.; Boerwinkle, E. dbNSFP v3.0: A one-stop database of functional predictions and annotations for human nonsynonymous and splice-site SNVs. *Hum. Mutat.* **2016**, *37*, 235–241. doi:10.1002/humu.22932.
5. Venselaar, H.; Beek, T.A.t.; Kuipers, R.K.P.; Hekkelman, M.L.; Vriend, G. Protein structure analysis of mutations causing inheritable diseases. An e-Science approach with life scientist friendly interfaces. *BMC Bioinformatics* **2010**, *11*, 548.
6. Schwarz, J.M.; Cooper, D.N.; Schuelke, M.; Seelow, D. MutationTaster2: Mutation prediction for the deep-sequencing age. *Nat. Publ. Gr.* **2014**, *11*, 361–362. doi:10.1038/nmeth.2890.
7. Choi, Y.; Sims, G.E.; Murphy, S.; Miller, J.R.; Chan, A.P. Predicting the functional effect of amino acid substitutions and indels. *PLoS ONE* **2012**, *7*, e46688. doi:10.1371/journal.pone.0046688.
8. Choi, Y.; Chan, A.P. PROVEAN web server: A tool to predict the functional effect of amino acid substitutions and indels. Bioinformatics **2015**, *31*, 2745–2747. doi:10.1093/bioinformatics/btv195.
9. Adzhubei, I.A.; Schmidt, S.; Peshkin, L.; Ramensky, V.E.; Gerasimova, A.; Bork, P.; Kondrashov, A.S.; Sunyaev, S.R. A method and server for predicting damaging missense mutations. *Nat. Publ. Gr.* **2010**, *7*, 248–249. doi:10.1038/nmeth0410-248.

10. Sim, N.-L.; Kumar, P.; Hu, J.; Henikoff, S.; Schneider, G.; Ng, P.C. SIFT web server: Predicting effects of amino acid substitutions on proteins. *Nucleic Acids Res.* **2012**, *40*, 452–457. doi:10.1093/nar/gks539.
11. Hu, J.; Ng, P.C. Predicting the effects of frameshifting indels. *Genome Biol.* **2012**, *12*, R9.
12. Hu, J.; Ng, P.C. SIFT indel: Predictions for the functional effects of amino acid insertions/deletions in proteins. *PLoS ONE* **2013**, *8*, e77940. doi:10.1371/journal.pone.0077940.
13. Douville, C.; Masica, D.L.; Stenson, P.D.; Cooper, D.N.; Gygax, D.M.; Kim, R.; Ryan, M.; Karchin, R. Assessing the pathogenicity of insertion and deletion variants with the variant effect scoring tool (VEST-indel). *Hum. Mutat.* **2016**, *37*, 28–35. doi:10.1002/humu.22911.
14. Desmet, F.-O.; Hamroun, D.; Lalande, M.; Collod-Béroud, G.; Claustres, M.; Béroud, C. Human splicing finder: An online bioinformatics tool to predict splicing signals. *Nucleic Acids Res.* **2009**, *37*, 1–14. doi:10.1093/nar/gkp215.
15. Vega, A.I.; Perez-Cerda, C.; Desviat, L.R.; Matthijs, G.; Ugarte, M.; Perez, B. Functional analysis of three splicing mutations identified in the PMM2 gene: Toward a new therapy for congenital disorder of glycosylation type Ia. *Hum. Mutat.* **2009**, *30*, 795–803. doi:10.1002/humu.20960.

Table S4 – **Characterization of deceased PMM2-CDG patients**. Immune-related manifestations and other clinical features of PMM2-CDG deceased patients reported in the ImmunoCDGQ.

**CDG**	**PMM2-CDG**			
**Age of death**	6 mo-old	21 mo-old	2 yrs-old	4.5 yrs-old
**Age range at diagnosis**	0-11 mo-old	0-11 mo-old	0-11 mo-old	0-11 mo-old
**Gender**	Female	Male	Male	Male
**Infections associated with cause of death**	No<br>Other: Cardiorespiratory failure secondary to liver failure	Yes	Yes	Yes
**Infection profile**$^{\alpha}$	NR	**Severe:**<br>Liver	**Frequent/chronic:**<br>Cold/flu, otitis	**Frequent/chronic:**<br>Eye, lung<br>**Severe:**<br>Blood
**Allergy profile**	NR	NR	Food allergies	NR
**Autoimmune disease**	NR	NR	NR	NR
**Frequent unexplained fever episodes**	Yes	No	No	Yes
**Vaccination**	Never vaccinated as recommended by HCP	Not taken all vaccines according to HCP recommendation and parents' decision.	Vaccinated. No relevant ADR reported.	Vaccinated. No relevant ADR reported.

		No relevant ADR reported		
**Laboratorial alterations**	**Sporadic:** High IGs, lymphopenia, neutrophilia[β]	**Constant:** HGG[μ] **Only during infections:** Leukocytosis	NR	**Sporadic:** Leukocytosis, leukopenia
**Other phenotypic features**	**Neurologic manifestations** HYP, ID, MC **GI/liver manifestations** HEPM/HEPSPL, high TRANS, CD, GR, Ftube, **Other** PE, TP, PA	**Neurologic manifestations** HYP, SZ, ID **GI/liver manifestations** High TRANS, GR **Other** PE, CM, PN, NS	**Neurologic manifestations** HYP, SZ **GI/liver manifestations** Ftube, CD **Other** CM	**Neurologic manifestations** HYP, SZ, ID **GI/liver manifestations** HEPM/HEPSPL, high TRANS, CD, PLE **Hematologic manifestations** TP, DWH, BT, PA **Other** PE, PN, NS
**Phenotype severity**	Very severe	Very severe	Severe	Severe

Legend: ADR: Adverse reactions; BT: Bruising tendency; CD: – Chronic diarrhea; CM: Cardiomyopathy; DWH: Delayed wound healing; Ftube: Feeding tube; GR: Gastroesophageal reflux; HEPM/HEPSPL: Hepatomegaly/hepatosplenomegaly; HCP: Healthcare professional; HGG: Hypogammaglobulinemia; HYP: Hypotonia; ID: Intellectual disability; IG: Immunoglobulin; MC: Microcephaly; mo: Months; NR: Not reported; NS: Nephrotic syndrome; PA: Persistent anemia; PE: Pericardial effusion; PLE: Protein-losing enteropathy; PN: Proteinuria; SLE: Stroke-like episodes; SZ: Seizures; TP: Thrombocytopenia; TRANS: Transaminases; yrs: Years

[α]In terms of infections, these patients were mainly reported to have suffered from severe infections and/or infections triggering other clinical manifestations rather than recurrent infections. Indeed, two of these patients were reported to have had permanent damage to their lungs and airways and liver resulting from infections.

[β]This patient had elevated IgG and IgA levels in the last tests, low T cells (CD3, CD4, CD8), high counts of CD3/DR (absolute counts too), $CD16^{+}56^{+}$ (NK cells), CD19 (B cells) and an increased CD4/8 an high ratio can be indicative of autoimmune disease). Indeed, in the autoimmune section the respondent reported suspicion of eosinophilic esophagitis. Additionally, low interferon production in response to phytohemagglutinin (PHA) and high production of lymphotoxin (LT) in response to PHA and Concanovalin A (ConA). Low total complement activity, with normal levels of C3 and C4. Neutrophilic bactericidal activity was low but neutrophil oxidative burst was normal.

[μ]Gammaglobulin deficit diagnosed within the first 28 days of life and requiring weekly IG injections (5 gr/weekly applied subcutaneously).

Table S5 – **PMM2-CDG patients with confirmed autoimmune manifestations.** Clinical features, autoimmune diseases and other immune-related manifestations described in the 3 PMM2-CDG patients with confirmed autoimmune manifestations.

**CDG**	**PMM2-CDG**		
**Age (current)**	11-20 yrs	11-20 yrs	4-10 yrs
**Age range at diagnosis**	0-11 mo	0-11 mo	6-10 yrs
**Gender**	Male	Female	Male
**Autoimmune disease**	Glomerulonephritis and celiac disease. Moderately improved with age. Autoimmune crisis associated with infection.	Celiac disease. Aggravated with age.	Inflammatory bowel disease. Slightly improved with age. Autoimmune crisis associated with infection.
**Autoimmune disease impact in QoL**	Overall impact: Worsens other CDG signs/symptoms<br>Everyday tasks: Extremely negative<br>Perceived autoimmune severity: Severe	Overall impact: Slightly negative<br>Everyday tasks: Slightly negative<br>Perceived autoimmune severity: Mild	Overall impact: Negative<br>Everyday tasks: Extremely negative<br>Perceived autoimmune severity: Severe
**Infection profile**	**Frequent/chronic:**<br>RT, lung, kidney, blood, and skin	**Frequent/chronic:**<br>Cold/flu and otitis	**Frequent/chronic:**<br>Cold/flu, RT, lung and GI tract
**Allergy profile**	Asthma, rhinitis, sinusitis and food allergies. Did not improve with age.	Food and drug allergies. Did not improve with age.	Drug allergies and hives. Resolved with age.
**Frequent unexplained fever episodes**	Yes	No	No
**Vaccination**	Vaccinated.<br>Vaccination ineffective due to loss of/failure to generate protective antibodies	Vaccinated.<br>No relevant ADR reported.	Never vaccinated. HCP not recommended vaccination.

**Laboratorial alterations**	**Present when younger:** HGG, high IG.	**Present when younger:** Lymphocytosis, neutrophilia, basophilia.	**Constant:** Leukocytosis.
**Other phenotypic features**	**Neurologic manifestations** HYP, SZ, ID **GI/liver manifestations** HEPM/HEPSPL, High TRANS, GR, Ftube, CD **Other** PE, OP, DWH, PA, PN, NS.	**Neurologic manifestations** HYP, SLE, ID **GI/liver manifestations** HEPM/HEPSPL, high TRANS **Other** PE, CM, OP, PN	**Neurologic manifestations** ID, HYP **Other** High TRANS, ICT, OP, TP, BT, BI, GR
**Phenotype severity**	Very severe	Moderate	Severe

Legend: ADR – Adverse reactions; BI – Behauviour issues; BT – Bruising tendency; CD – Chronic diarrhea; CM – Cardiomyopathy; DWH – Delayed wound healing; Ftube – Feeding tube; GI – Gastrointestinal; GR – Gastroesophageal reflux; HEPM/HEPSPL – Hepatomegaly/hepatosplenomegaly; HCP – Healthcare professional; HGG – Hypogammaglobulinemia; HYP – Hypotonia; ICT – Ichthyosis; ID – Intellectual disability; IG – Immunoglobulin; mo – Months; NS – Nephrotic syndrome; OP – Osteopenia/osteoporosis; PA – Persistent anemia; PE – Pericardial effusion; PN – Proteinuria; QoL – Quality of life; RT – Respiratory tract; SLE – Stroke-like episodes; SZ – Seizures; TP – Thrombocytopenia; TRANS – Transaminases; yrs – Years

Table S6 – **Immune-related manifestations and other clinical data in less frequent CDG (non-PMM2-CDG group).** This table lists the non-PMM2-CDGs (n=34) reported in the ImmunoCDGQ organized by glycosylation mechanism.

CDG (#MIM number) (patients)	Mutation(s): Protein change	Age range (current)	*Immune-related manifestations*						
			Infection profile				Allergy profile	Autoimmunity profile	Laboratorial alterations
			Infections	Age range *(when infections most frequent/severe)*	Infectious agents *(prevalent)*	Treatment			
**N-glycosylation**									
ALG1-CDG[α] #608540 n=2 (1 M,1 F)	V281F/Y353D (1/2) NR (1/2)	13 mo-3 yrs (1/2) 11-20 yrs (1/2)	Relevant related issues (2/2) **Frequent/chronic:** Otitis, RT, lung, GI tract, skin (1/2)	Birth-3 yrs (1/2)	Bacterial (2/2)	Antibiotics (2/2)	**Allergies** (1/2): Food, drugs, dermatitis, eczema. Slightly improving with age.	**AD** (1/2): EoE, slightly improving with age	NR
ALG3-CDG #601110 n=3 (2 M,1 F)	NR	0-6 mo (1/3) 4-10 yrs (1/3) +20 yrs (1/3)	Relevant related issues (2/3) **Frequent/chronic:** Cold/flu, otitis, RT, lung (1/2)	6 mo-2 yrs (1/2) Birth-6 yrs (1/2)	Bacterial (1/2) Viral (1/2)	Antibiotics (1/2)	**Allergies** (1/3): Drug[β], hives, dermatitis, eczema. Did not improve with age	**AD** (1/3): Psoriasis not improving with age	**Constant:** Leukocytosis (1/3)
ALG6-CDG[γ] #603147 n= 9 (6 M,3 F)	A333V/ c.495-2A>G (1/9) A333V/A333V (1/9)	7-12 mo (1/9) 13 mo-3 yrs (1/9) 4-10 yrs (2/9) 1-20 yrs (2/9)	Relevant related issues (9/9) **Frequent/ chronic:** Cold/flu, RT (6/9); otitis, lung (3/9); eye, GI tract (2/9);	Birth-3 yrs (6/9) Birth-6 yrs (2/9) Unknown (1/9)[κ]	Viral (5/9) Bacterial (3/9) Unknown (1/9)	Antibiotics (9/9): Ineffective (1/9) IVIG (1/9)	**Allergies** (1/9): Asthma, sinusitis, eczema Allergic episodes	**AD** (1/9): Psoriasis slightly improving with age.	**Constant:** Lymphocytosis, neutropenia, monocytosis (1/9) **Present when**

CDG (#MIM number) (patients)	Mutation(s): Protein change	Age range (current)	***Immune-related manifestations***						
			Infection profile				Allergy profile	Autoimmunity profile	Laboratorial alterations
			Infections	Age range *(when infections most frequent/severe)*	Infectious agents *(prevalent)*	Treatment			
	A333V/ c.257+5G>A (1/9)<br>L452R/ c.257+5G>A (1/9)<br>NR (5/9)	+20 yrs (3/9)	tooth/mouth (1/9)<br>**Severe:**<br>Blood (2/9)			Immune modulators (1/9): Ineffective	associated with infections and improved with age.		**younger:**<br>HGG, DGG, leukopenia, basophilia, monocytosis (1/9)
ALG8-CDG #608104 n=3 (2 M,1 F)	NR	4-10 yrs (2/3)<br>11-20 yrs (1/3)	Relevant related issues (2/3)<br>**Frequent/chronic:**<br>Cold/flu, UTI (1/2)	Birth-6 yrs (1/2)<br>Unknown (1/2)	Viral (1/2)<br>No preponderant agent[ð] (1/2)	Antibiotics (2/2)	**Allergies** (2/3): Asthma, rhinitis, sinusitis, food, hives, dermatitis (1/2). Improved with age: moderately (1/2) greatly (1/2)	NR	**Present when younger:**<br>High Ig (1/3)

New insights into immunological involvement in Congenital Disorders of Glycosylation (CDG) from a people-centric approach

Supplementary Material

CDG (#MIM number) (patients)	Mutation(s): Protein change	Age range (current)	*Immune-related manifestations*						
			Infection profile				Allergy profile	Autoimmunity profile	Laboratorial alterations
			Infections	Age range *(when infections most frequent/severe)*	Infectious agents *(prevalent)*	Treatment			
ALG9-CDG #608776 n=3 (1 M,2 F)	E352K/E352K (1/3) Y287C/Y287C (1/3) NR (1/3)	4-10 yrs (1/3) 11-20 yrs (2/3)	Relevant related issues (3/3) **Frequent and/or chronic:** Cold/flu (2/3); RT, GI tract (1/3) **Severe:** Blood (1/3)	Birth-3 yrs (2/3) Unknown (1/3)	Viral (1/3) Unknown (2/3)	Antibiotics (2/3) IVIG (1/3): Ineffective	NR	NR	**Constant:** DGG, high Ig (1/3)
ALG11-CDG #613661 n=3 (1 M,2 F)	Q318P/Q318P (1/3) E312G/M408R (1/3) L31*/ A85V (1/3)	13 mo-3 yrs (1/3) 4-10 yrs (1/3) 11-20 yrs (1/3)	NR				Sinusitis (1/3)	NR	**Constant:** Leukocytosis, neutrophilia (1/3)
ALG12-CDG #607143 n=3 (2 M,1 F)	A81T7/A81T (1/3) G123R/L480P (1/3) NR (1/3)	4-10 yrs (2/3) 11-20 yrs (1/3)	Relevant related issues (2/3) **Frequent and/or chronic:** Cold/flu (2/2); otitis, RT (1/2)	Birth-3 yrs (1/2) Birth-6 yrs (1/2)	Viral (1/2) Unknown (1/2)	Antibiotics (2/2)	**Allergies** (2/3): Sinusitis, dermatitis, eczema (1/2) Did not improve with age (1/2)	NR	NR

New insights into immunological involvement in Congenital Disorders of Glycosylation (CDG) from a people-centric approach

Supplementary Material

CDG (#MIM number) (patients)	Mutation(s): Protein change	Age range (current)	*Immune-related manifestations*						
			Infection profile				Allergy profile	Autoimmunity profile	Laboratorial alterations
			Infections	Age range *(when infections most frequent/severe)*	Infectious agents *(prevalent)*	Treatment			
							Slightly improved with age (1/2)		
ALG13-CDG[γ] #300884 n=5 (2 M,3 F)	N107S (2/5) NR (3/5)	7-12 mo (1/5) 13 mo-3 yrs (1/5) 4-10 yrs (2/5) 11-20 yrs (1/5)	Relevant related issues (1/5) **Frequent and/or chronic:** Cold/flu, RT, lung	Birth-3 yrs	Bacterial	Antibiotics	**Allergies** (3/5): Eczema (3/3), rhinitis (1/3). Did not improve with age (1/3) Moderately improved with age (1/3)	NR	**Present when younger:** HGG (1/5)
DPAGT1-CDG[α] #191350 n=3 (3 F)	R247W/ M9Ifs*80 (1/3) NR (2/3)	11-20 yrs (3/3)	Relevant related issues (2/3) **Frequent and/or chronic:** Cold/flu, RT, lung (1/2) **Severe:** Blood (1/2)	4-6 yrs (1/2) 7-10 yrs (1/2)	No preponderant agent (2/2)	Antibiotics (2/2): Ineffective (1/2)	NR	NR	NR
DPM1-CDG[α] #608799 n=1, M	NR	+20 yrs	Relevant related issues **Frequent/chronic:** RT **Severe:**	Birth-3 yrs	Bacterial[ϱ]	Antibiotics	NR	NR	NR

CDG (#MIM number) (patients)	Mutation(s): Protein change	Age range (current)	*Immune-related manifestations*						
			Infection profile				Allergy profile	Autoimmunity profile	Laboratorial alterations
			Infections	Age range *(when infections most frequent/severe)*	Infectious agents *(prevalent)*	Treatment			
			Blood						
FUT8-CDG #618005 n=1, F	NR	Deceased[†](40 mo)	Relevant related issues (1/1) **Frequent/chronic:** Cold/flu, lungs[σ]	Birth-3 yrs	Viral	Antibiotics: Ineffective	NR	NR	NR
MAN1B1-CDG #614202 n=1, M	NR	4-10 yrs	Relevant related issues (1/1) **Frequent/chronic:** Cold/flu, otitis, RT	Unknown	No preponderant agent (1/1)	Antibiotics	Food, did not improve with age.	Celiac disease did not improve with age.	NR
MPDU1-CDG #609180 n=1, M	NR	4-10 yrs	NR	NR	NR	NR	NR	NR	NR
MPI-CDG[α] #602579 n=3 (2 M,1 F)	P76L/P76L (1/3) NR (2/3)	4-10 yrs (2/3) +20 yrs (1/3)	Relevant related issues (3/3) **Frequent/chronic:** Cold/flu, GI tract (3/3); RT (1/3) **Severe:** Brain, blood (1/3)	Birth-3 yrs (2/3) Birth-10 yrs (1/3)	Bacterial (1/3) Viral (1/3) Unknown (1/3)	Antibiotics (3/3): Ineffective (1/3)	**Allergies** (1/3): Anesthesia greatly improved with age	NR	**Present when younger:** Leukocytosis, leukopenia (1/3)

New insights into immunological involvement in Congenital Disorders of Glycosylation (CDG) from a people-centric approach

Supplementary Material

CDG (#MIM number) (patients)	Mutation(s): Protein change	Age range (current)	*Immune-related manifestations*							
			Infection profile				Allergy profile	Autoimmunity profile	Laboratorial alterations	
			Infections	Age range *(when infections most frequent/severe)*	Infectious agents *(prevalent)*	Treatment				
MOGS-CDG<br>#606056<br>n=3 (1 M,2 F)	R561C/ E295Nfs*10 (1/3)<br>Q124*/E487D (1/3)<br>P513S/G824D (1/3)	13 mo-3 yrs (1/3)<br>11-20 yrs (1/3)<br>Deceased [†](1/3) (3 mo)	Relevant related issues (3/3)<br>**Frequent/chronic:**<br>Lung (3/3); RT, GI tract, kidney, blood (2/3); cold/flu, liver, skin (1/3)	Birth-3 yrs (1/3)<br>4-6 yrs (1/3)<br>Unknown[ς] (1/3)	Bacterial[λ] (3/3)	Antibiotics (3/3): Ineffective (developed resistance) (2/3).<br>IVIG: Ineffective (2/3)<br>Immune modulators: Ineffective (1/3)	**Allergies** (1/3): Sinusitis associated with infections did not improve with age	NR	**Constant:**<br>Leukocytosis (2/3)<br>Lymphocytosis, HGG, DGG, High Ig (1/3)<br>**Sporadic:**<br>DGG, Eosinophilia (1/3)<br>**Present when younger:**<br>Lymphopenia (1/3) [ϑ]	
SRD5A3-CDG<br>#612379<br>n=4 (1 M,3 F)	L178P/ L178P (1/4)<br>W201*/ W201* (1/4)<br>NR (2/4)	4-10 yrs (2/4)<br>11-20 yrs (2/4)	Relevant related issues (1/4)[ч]	Birth-3 yrs	Unknown	Antibiotics (1/1)	**Allergies** (3/4): Eczema (3/3); asthma, sinusitis, dermatitis (1/3)<br>Did not improved with age (1/3)<br>Greatly improved with age (1/3)	NR	NR	
SLC39A8-CDG[γ]<br>#616721<br>n=2 (2 M)	NR	13 mo-3 yrs (1/2)<br>4-10 yrs (1/2)	Relevant related issues (1/2)<br>**Frequent/chronic:**<br>RT, lungs	7-10 yrs	Bacterial[×]	Antibiotics and IVIG	Asthma (1/2)	NR	NR	

CDG (#MIM number) (patients)	Mutation(s): Protein change	Age range (current)	*Immune-related manifestations*						
			Infection profile				Allergy profile	Autoimmunity profile	Laboratorial alterations
			Infections	Age range *(when infections most frequent/severe)*	Infectious agents *(prevalent)*	Treatment			
TMEM165-CDG[γ] #614727 n=1, M	NR	11-20 yrs	NR	NR	NR	NR	NR	Eczema Greatly improved with age.	NR
				**O- glycosylation**					
OGT-CDG[ε] #300997 n=1, M	N648Y	4-10 yrs	Relevant related issues **Frequent/chronic:** Cold/flu, GI tract, RT, otitis	Birth-3 yrs	No preponderant agent	Antibiotics: Ineffective	Anesthesia, food, sinusitis Associated with infections moderately improved with age.	NR	NR
				**GPI glycosylation**					
PIGA-CDG[γ] #300868 n=4 (4 M)	S132C (2/4) NR (2/4)	13 mo-3 yrs (1/4) 4-10 yrs (3/4)	Relevant related issues (4/4) **Frequent and/or chronic**:	Birth-3 yrs (3/4)	Viral (4/4)	Antibiotics (4/4):	Allergies (4/4): Eczema (2/4)	NR	**Sporadic:** Basophilia (3/4) Eosinophilia (2/4)

New insights into immunological involvement in Congenital Disorders of Glycosylation (CDG) from a people-centric approach

Supplementary Material

CDG (#MIM number) (patients)	Mutation(s): Protein change	Age range (current)	*Immune-related manifestations*						
			Infection profile				Allergy profile	Autoimmunity profile	Laboratorial alterations
			Infections	Age range *(when infections most frequent/severe)*	Infectious agents *(prevalent)*	Treatment			
			Otitis (3/4), cold/flu, lung (2/4); RT (1/4) **Severe:** Blood (1/4)	Birth-6 yrs (1/4)		Ineffective (2/4)	Asthma, Food, Hives (1/4) Related to infections (1/4) Did not improve with age (1/4) Improved with age: Moderately (1/4) Greatly (1/4)		High Igs (2/4)[ϰ] Lymphopenia (1/4) Neutrophilia (1/4) Neutropenia (1/4) Monocytosis (1/4) Monocytopenia (1/4)
PIGN-CDG #614080 n=2 (2 F)	NR	4-10 yrs (1/2) 11-20 yrs (1/2)	Relevant related issues (2/2) **Frequent/chronic:** Cold/flu, RT (1/2)	Birth-3 yrs (1/2) Unknown (1/2)	Viral (1/2) Bacterial (1/2)	Antibiotics (2/2) Ineffective (1/2)	Allergies (1/2): food, drugs, hives	HA (1/2)	**Present when Younger:** Leukopenia, neutrophilia (1/2)
PIGT-CDG #615398 n=1, M	NR	11-20 yrs	Relevant related issues **Frequent/chronic:** Lungs **Severe:** Blood	Birth-6 yrs	Bacterial	Antibiotics and IVIG	Hives Associated with infections and slightly improved with age.	NR	**Constant:** DGG[θ]

New insights into immunological involvement in Congenital Disorders of Glycosylation (CDG) from a people-centric approach

Supplementary Material

CDG (#MIM number) (patients)	Mutation(s): Protein change	Age range (current)	*Immune-related manifestations*							
			Infection profile				Allergy profile	Autoimmunity profile	Laboratorial alterations	
			Infections	Age range *(when infections most frequent/severe)*	Infectious agents *(prevalent)*	Treatment				
**Glycosaminoglycans/proteoglycans glycosylation**										
B4GALT7-CDG[ɛ] #130070 n=2 (2 F)	H93Y/R62K (2/2)	11-20 yrs (2/2)	Relevant related issues (2/2) **Frequent/chronic:** Otitis, eye, tooth/mouth (2/2) **Severe:** Brain (2/2)	Birth-10 yrs (2/2)	There is no preponderant agent[ʔ] (2/2)	Antibiotics (2/2)	Allergies (2/2): drugs, asthma, dermatitis, eczema (2/2) associated with infections (2/2).	NR[ϐ]	**Constant:** Leukopenia, neutropenia[ϕ] (2/2) **Sporadic:** DGG, high IG, lymphopenia (2/2)	
**Multiple pathways**										
COG1-CDG #611209 n=1, M	NR	4-10 yrs	Relevant related issues **Frequent/chronic:** GI tract	Birth-3 yrs	Viral	Antibiotics: Ineffective	Food and drugs. Associated with infections and slightly improved with age.	NR	**Present when Younger:** Leukopenia	
COG4-CDG #613489 n=1, M	NR	11-20 yrs	Relevant related issues **Frequent/chronic:** Cold/flu, lung, RT, GI, skin	Birth-3 yrs	Viral	Antibiotics and immune modulators	NR	NR	**Constant:** Leukopenia, neutropenia[ю] **Present when Younger:** Monocytosis, eosinophilia	

New insights into immunological involvement in Congenital Disorders of Glycosylation (CDG) from a people-centric approach

Supplementary Material

CDG (#MIM number) (patients)	Mutation(s): Protein change	Age range (current)	*Immune-related manifestations*						
			Infection profile				Allergy profile	Autoimmunity profile	Laboratorial alterations
			Infections	Age range *(when infections most frequent/severe)*	Infectious agents *(prevalent)*	Treatment			
COG5-CDG #613612 n=3 (3 M)	NR (3/3)	13 mo-3 yrs (1/3) $^{\gamma}$ 11-30 yrs (1/3) +20 yrs (1/3)	Relevant related issues (1/3) **Frequent and/or chronic:** Cold/flu, RT, UTI	Birth-3 yrs	Unknown	Antibiotics	Rhinitis (1/3)	NR	NR
COG6-CDG #614576 n=3 (1 M,2 F)	c.1167-24A>G/ c.1167-24A>G (2/3) NR (1/3)	11-20 yrs (1/3) +20 yrs (2/3)	NR	NR	NR	NR	NR	Eczema slightly improved with age (1/3)	NR

New insights into immunological involvement in Congenital Disorders of Glycosylation (CDG) from a people-centric approach

Supplementary Material

CDG (#MIM number) (patients)	Mutation(s): Protein change	Age range (current)	*Immune-related manifestations*						
			Infection profile				Allergy profile	Autoimmunity profile	Laboratorial alterations
			Infections	Age range *(when infections most frequent/severe)*	Infectious agents *(prevalent)*	Treatment			
COG8-CDG[α] #611182 n=1, F	NR	+20 yrs	Relevant related issues **Frequent/chronic:** Cold/flu, RT, otitis, tooth/mouth[⁎] **Severe:** Blood	Birth-10 yrs	No preponderant agent	Antibiotics	NR	NR	NR
GMPPA-CDG #615510 n=2 (2 M)	NR	13 mo-3 yrs (1/2) 4-10 yrs (1/2)	NR	NR	NR	NR	NR	NR	NR
GMPPB-CDG[α] #15352 n=1, F	NR	11-20 yrs	Relevant related issues (1/1) **Frequent/chronic:** Cold/flu, GI tract	Unknown	Viral	Antibiotics Rest and fluids (mild viral infections)	Dermatitis, eczema, rhinitis, grass and pollen not improved with age	NR	NR[u]
NANS-CDG #610442 n=2 (2 M)	G31Afs*5/ R237H (1/2) R237C/Y188H (1/2)	4-10 yrs (1/2) +20 yrs (1/2)	Relevant related issues (2/2) **Frequent/chronic:** RT (2/2), cold/flu, otitis, GI tract, kidney, bladder/UTI (1/2)	4 -6 yrs (1/2) Unknown (1/2)	No preponderant agent (1/2) Unknown (1/2)	Antibiotics (1/2) IVIG: Ineffective (1/2)[χ]	**Allergies** (1/2): Dermatitits, drugs, food, rhinitis did not improve with age	NR	**Constant:** Neutropenia (1/2) **Sporadic:** DGG (1/2)[χ]

New insights into immunological involvement in Congenital Disorders of Glycosylation (CDG) from a people-centric approach

Supplementary Material

CDG (#MIM number) (patients)	Mutation(s): Protein change	Age range (current)	*Immune-related manifestations*						
			Infection profile				Allergy profile	Autoimmunity profile	Laboratorial alterations
			Infections	Age range *(when infections most frequent/severe)*	Infectious agents *(prevalent)*	Treatment			
PGM1-CDG[γ] #614921 n=4 (2 M,2 F)	R503*/ G554* (1/4) NR (3/4)	4-10 yrs (3/4) Deceased[†] (4 mo) (1/4)	Relevant related issues (2/4) **Frequent and/or chronic:** Otitis (1/2) **Severe**[w]: Blood infection (1/2)	Birth-3 yrs (1/2) Unknown[£] (1/2)	Bacterial (1/2) Unknown[£] (1/2)	Antibiotics: Ineffective (1/2)	**Allergies** (2/4): Drug, dermatitis (1/2) Did not improve with age (1/2) Slightly improved with age (1/2)	NR	**Sporadic:** Lymphopenia, monocytosis (1/4) **Present when younger** DGG (1/4)
SLC35A2-CDG[γ] #300896 n=7 (3 M,4 F)	M1K (1/7) L224F (1/7) R58Pfs*36 (1/7) NR (4/7)	13 mo-3 yrs (4/7) 4-10 yrs (1/7) 11-20 yrs (2/7)	Relevant related issues (4/7) **Frequent and/or chronic:** RT (2/4); cold/flu, lung, eye, tooth/mouth, GI tract, UTI/kidney (1/4)	Birth-3 yrs (2/4) 4 -6 yrs (2/4)	Viral (3/4) No preponderant agent (1/4)	Antibiotics (4/4): Ineffective (1/4) IVIG (1/4)	**Allergies** (3/7): Drugs, rhinitis, sinusitis, eczema, pollen (1/3) related to infections (1/3). Not improved with age (1/3) improved with age: slightly (1/3) moderately (1/3)	NR	**Sporadic:** Leukocytosis (1/7) Lymphocytosis (1/7)
SLC35C1-CDG #266265 n=1, F	NR	11-20 yrs	NR	NR	NR	NR	NR	NR	NR

New insights into immunological involvement in Congenital Disorders of Glycosylation (CDG) from a people-centric approach

Supplementary Material

Legend: AD – Autoimmune diseases; ADR – Adverse reactions; ART – Arthritis, arthralgia or joint contractures; BI - Behaviour issues; BT – Bruising tendency; CD -Chronic diarrhea; CM – Cardiomyopathy; DGG – Dysgammaglobulinemia; DWH - Delayed wound healing; EoE – Eosinophilic esophagitis; F – Female; FTube – Feeding tube; GI – Gastrointestinal; GR – Gastroesophageal reflux; HA – Hemolytic anemia; HCP – Healthcare professional; HEPM/HEPSPL – Hepatomegaly/hepatosplenomegaly; HGG – Hypogammaglobulinemia; HYP – Hypotonia; ICT – Ichthyosis; ID -Intellectual disability; IG – Immunoglobulin; IVIG – Intravenous immunoglobulins; IFEB – Inability to fully empty the bladder; LAIR – Lung and airways; M - Male; MC – Microcephaly; mo – Months; NR – Not reported; NS – Nephrotic syndrome; OP – Osteopenia/osteoporosis; PA – Persistent anemia; PE – Pericardial effusion; PLE – Protein-losing enteropathy; PN – Proteinuria; RT – Respiratory tract; SLE - Stroke-like episodes; SZ – Seizures; TP - Thrombocytopenia; TRANS – Transaminases; UTI – Urinary tract infections; yrs – Years

[α]Mannose supplementation in one ALG1-CDG, a COG8-CDG, a DPM1-CDG and a DPAGT1-CDG patient had no effect on immune-related manifestations. However, two MPI-CDG and a GMPPB-CDG patients described mannose supplementation to have improved immunological manifestations.

[β]Allergies to the antibiotic Sulfamethoxazole with trimethoprim (Bactrim®, Septra®). Additionally, allergies (hives) to the *Haemophilus influenzae* type B vaccine (HIB), Diphtheria, Tetanus, Pertussis (DTaP), Pneumococcal conjugate vaccine (PCV13) vaccines reported for this patient.

[γ] Galactose supplementation in two ALG6-CDG and one ALG13-CDG patients reportedly had no effect on immune-related manifestations. In a PIGA-CDG patient it reportedly worsened immune issues, while in one COG5-CDG, a SLC35A2-CDG, a SLC39A8-CDG and a TMEM165-CDG patients it improved immune problems. Additionally, PGM1-CDG patients reported galactose supplementation to have improved/corrected all immunological issues/dysfunction.

[κ]This was reported for the patient aged 7-12 months old.

[δ]Regarding most common infectious agents: *E. coli* or *Morganella morganii* for UTI, presumed viral agent for RT infections.

[ϱ]*Streptococcus pneumoniae* stated as the most common infectious agent.

[†]In the cases of FUT8-CDG and MOGS-CDG deceased patients, infection was among the cause of death. Contrarily, in the deceased PGM1-CDG, a blood clot was the cause of death.

[σ]Lung infection reported for this patient was recurrent bronchiolitis.

[π]Severe seizures after measles, mumps and rubella (MMR) vaccination.

[ς]This MOGS-CDG patient died at the age of 3 months. He was reported to have suffered from frequent/severe infections during his whole life.

[λ]*Mycoplasma* as a bronchopneumonia-causing agent was identified as the most common infectious agent in one MOGS-CDG patient. This patient had been taking phenobarbital and the respondents associated the withdrawal of this drug with an improvement of these atypical *Mycoplasma*-derived infections.

[ϑ]A MOGS-CDG patient was described to have presented low CD4, CD8 and CD18, which normalized with age.

[ω]This MOGS-CDG patient has only taken the hepatitis and Bacillus Calmette–Guérin (BCG) vaccines, failing to respond to the latter.

[ϥ]This patient is not reported to have had a specific frequent/chronic infection(s). However, the infections which were most frequently reported – both in the past and present – are cold/flu, RT and lung infections.

[ˣ]*Mycoplasma* was the most identified infectious agent, responsible for the RT/lung infections.

[ε]Magnesium supplementation improved immune-related issues in two B4GALT7-CDG patients and in an OGT-CDG patient. Additionally, the OGT-CDG patient reported that manganese and GlcNAc supplementation also led to an immunological improvement.

[ϰ]In two PIGA-CDG patients, sporadically elevated IgE levels, basophils and eosinophils (both % and absolute values) were reported.

[θ]PIGT-CDG patient reported to have IgA deficiency.

[ƨ]Bacteria belonging to the *Pseudomonas* and *Staphylococcus genus*. As for virus, respondent reported common virus and Epstein-Barr virus (EBV). Respondent added that viral-induced secondary bacterial infections happen frequently.

[б]Respondent reported elevated ANA levels, but no definitive diagnosis made.

[ф]Constant neutropenia and leukopenia (mild) associated with bone marrow failure.

[ю]COG4-CDG patient reported to take Granulocyte-colony stimulating factor (G-CSF), which according to respondent has improved infections.

[ж]The respondent highlighted the recurrent aphthous stomatitis, which has been a continuous problem throughout the patients' entire life.

[U]Although no concrete laboratorial alteration was pinpointed the respondent made the following comment "At points in time, many of these tests have been odd, high eosinophils and varying levels of RBC and WBC, but nothing systemic or persistent."

[ж]IVIG in the NANS-CDG patient has been used mainly to see if it could improve low platelet counts. However, sporadic low IgG and secretory IgA have been found.

[w]In PGM1-CDG, both patients described to have relevant issues associated with infections were reported to mainly suffer from severe infections.

[Ⴖ]This was replied by the respondent reporting the PGM1-CDG patient deceased at 4 months-old.

[z] Patient on adrenocorticoid therapy (ACTH). Patients on ACTH therapy are recommended not to receive some vaccines, namely live virus vaccines.

Table S7 – **Characteristics of the control, PMM2-CDG and non-PMM2-CDG groups with relevant infections.** Age range and gender distribution among the study groups who reported relevant infections.

		Control	PMM2-CDG	non-PMM2-CDG[4]
**Age range**	0-6 mo	1.9 % (n=1/53)	0 %	1.8 % (n=1/57)
	7-12 mo	0 %	1.5% (n=1/65)	1.8 % (n=1/57)
	13 mo–3 yrs	7.6 % (n=4/53)	9.2% (n=6/65)	10.5 % (n=6/57)
	4-10 yrs	24.5 % (n=13/53)	43.1% (n=28/65)	38.6 % (n=22/57)
	11-20 yrs	7.6 % (n=4/53)	18.5% (n=12/65)	28.1 % (n=16/57)
	+20 yrs	58.5 % (n=31/53)	23.1% (n=15/65)	14.0 % (n=8/57)
	Deceased[1]	NA	4.6% (n=3/65)	5.3 % (n=3/57)
**Gender**	Male	22.6 % (n=12/53)[2]	47.7% (n=31/65)[3]	59.7 % (n=34/57)[5]
	Female	77.4 % (n=41/53)[2]	52.3% (n=34/65)[3]	40.4 % (n=23/57)[5]

Legend: NA – Not applicable

[1]PMM2-CDG deceased patients were 21 months, 2 years and 4.5 years-old at the time of death

[2]When the control group with relevant infections is considered there is a higher prevalence of females. However, if we calculate infection prevalence for each gender – based on the total number of males and females (males: 11.4 %, n=41/105; females: 16.8 %, n= 117/244) infection-relevant issues is gender-balanced and consistently low.

[3]In the PMM2-CDG group with relevant infections, gender distribution is balanced. Similar relevant infection issues prevalence by gender is observed when we consider the entire PMM2-CDG cohort (males: 53.5 %, n=31/58; females: 53.1 %, n=34/64).

[4]CDG included in the non-PMM2-CDG sub-group with relevant infections: ALG1-CDG (n=2); ALG3-CDG (n=2); ALG6-CDG (n=9); ALG8-CDG (n=2); ALG9-CDG (n=3); ALG12-CDG (n=2); ALG13-CDG (n=1); B4GALT7-CDG (n=2); COG1-CDG (n=1); COG4-CDG (n=1); COG5-CDG (n=1); COG8-CDG (n=1); DPAGT1-CDG (n=2); DPM1-CDG (n=1); FUT8-CDG (n=1); GMPPB-CDG (n=1); MAN1B1-CDG (n=1); MOGS-CDG (n=3); MPI-CDG (n=3); NANS-CDG (n=2); OGT-CDG (n=1); PGM1-CDG (n=2); PIGA-CDG (n=4); PIGN-CDG (n=2); PIGT-CDG (n=1); SLC35A2 (n=4); SLC39A8 (n=1); SRD5A3 (n=1)

[5]In the non-PMM2-CDG group with relevant infections, the percentage of males is higher. This could be partly explained by the prevalence of X-linked CDG in this group. However, if we calculate infection prevalence for each gender – based on the total number of males and females (males: 69.4 %, n=34/49; females: 58.9 %, n=23/39) the gender trend - with more males presenting infection-relevant issues - is maintained.

Table S8 – **Characteristics of the control, PMM2-CDG and non-PMM2-CDG groups with allergies.** Age range and gender distribution among the study groups who reported relevant allergies

		Control	PMM2-CDG	non-PMM2-CDG[4]
**Age range**	0-6 mo	0.6 % (n=1/158)	0 %	0 %
	7-12 mo	1.3 % (n=2/158)	2.4 % (n=1/41)	0 %
	13 mo–3 yrs	3.8 % (n=6/158)	7.3 % (n=3/41)	10.5 % (n=4/38)
	4-10 yrs	17.7 % (n=28/158)	41.5 % (n=17/41)	44.7 % (n=17/38)
	11-20 yrs	6.9 % (n=11/158)	17.1 % (n=7/41)	34.2 % (n=13/38)
	+20 yrs	69.6 % (110/158)	29.3 % (n=12/41)	10.5 % (n=4/38)
	Deceased[1]	NA	2.4 % (n=1/41)	NA
**Gender**	Male	25.9 % (n=41/158)[2]	56.1 % (n=23/41)[3]	65.8 % (25/38)[5]
	Female	74.1 % (n=117/158)[2]	43.9 % (n=18/41)[3]	34.2 % (n=13/38)[5]

Legend: NA – Not applicable

[1]The PMM2-CDG deceased patient was 2 years-old at the time of death

[2]When we consider the allergic group in the control, there is a higher prevalence of females. However, if we consider the total number of males and females of the control samples, the percentage of participants from each gender is more balanced (males: 39.1 %, n=41/105; females: 47.9 %, n=117/244).

[3]In the PMM2-CDG group with allergies, there is a slightly higher male prevalence. The same allergy prevalence by gender is maintained when we consider the total number of PMM2-CDG males and females (males: 39.7 %, n=23/58; females: 28.1 %, n=18/64).

[4]CDG included in the non-PMM2-CDG group with allergies: ALG1-CDG (n=1); ALG3-CDG (n=1); ALG6-CDG (n=1); ALG8-CDG (n=2); ALG11-CDG (n=1); AL12-CDG (n=2); ALG13-CDG (n=3); B4GALT7-CDG (n=2); COG1-CDG (n=1); COG5 (n=1); COG6-CDG (n=1); GMPPB-CDG (n=1); MAN1B1-CDG (n=1); MOGS-CDG (n=1); MPI-CDG (n=1); NANS-CDG (n=1); OGT-CDG (n=1); PGM1-CDG (n=2); PIGA-CDG (n=4); PIGN-CDG (n=1); PIGT-CDG(n=1); SLC35A2-CDG (n=3); SLC39A8-CDG (n=1); SRD5A3-CDG (n=3); TMEM165-CDG (n=1).

[5]In the non-PMM2-CDG group with allergies, the percentage of males is much higher. This could be partly explained by the prevalence of X-linked CDG in this sub-sample. However, if we calculate infection prevalence for each gender – based on the total number of males and females (males: 51.0 %, n=25/49; females: 33.3 %, n=13/39) the trend - with more males presenting allergies - is maintained.

Table S9 – **Correlation of PMM2-CDG clinical features with infections and allergies in PMM2-CDG.** Clinical manifestations significantly associated with immune-related features are highlighted in green. Statistical significance was calculated with the

Fisher exact test and statistical significance was set at *p*-value ≤ 0.05. Legend: NA – Not available; NS – Not significant; OR – Odds ratio.

	Other clinical features	Relevant infections			Allergi	
		**W/ infections (n=65)**	**W/o infections (n=57)**	***p*-value/OR**	**W/ allergies (n=41)**	**W/o allergie**
**Liver**	Hepatomegaly/hepatosplenomegaly	33.9 % (n=22)	17.5 % (n=10)	NS/2.39	29.3 % (n=12)	24.7 % (n
	Elevated liver transaminases	64.6 % (n=42)	40.4 % (n=23)	0.011/2.68	60.9 % (n=25)	49.4 % (n
**Heart**	Pericardial effusion	27.7 % (n=18)	10.5 % (n=6)	0.022/3.23	19.5 % (n=8)	19.8 % (n
	Cardiomyopathy	10.8 % (n=7)	0 %	0.014/NA	9.8 % (n=4)	3.7 % (n
**Skin, joints and bone**	Ichthyosis	4.6 % (n=3)	0 %	NS/NA	4.9 % (n=2)	1.2 % (n
	Arthrosis/arthralgia/joint contractures	6.2 % (n=4)	7.0 % (n=4)	NS/0.870	12.2 % (n=5)	3.7 % (n
	Osteopenia/osteoporosis	32.3 % (n=21)	21.1 % (n=12)	NS/1.78	46.3 % (n=19)	17.3 % (n
**Blood**	Thrombocytopenia	15.4 % (n=10)	7.0 % (n=4)	NS/2.39	14.6 % (n=6)	9.9 % (n
	Delayed wound healing	15.4 % (n=10)	15.8 % (n=9)	NS/0.970	24.4 % (n=10)	11.1 % (
	Bruising tendency	29.2 % (n=19)	22.8 % (n=13)	NS/1.39	31.7 % (n=13)	23.5 % (n
	Persistent anemia	18.5 % (n=12)	10.5 % (n=6)	NS/1.91	12.2 % (n=5)	16.1 % (n
**Brain/Central nervous system**	Intellectual disability	76.9 % (n=50)	56.1 % (n=32)	0.020/2.58	70.7 % (n=29)	65.4 % (n
	Stroke-like episodes	44.6 % (n=29)	12.3 % (n=7)	0.00012/5.67	34.2 % (n=14)	27.2 % (n
	Microcephaly	12.3 % (n=8)	12.3 % (n=7)	NS/1.00	12.2 % (n=5)	12.4 % (n
	Hypotonia	95.4 % (n=62)	85.9 % (n=49)	NS/3.34	97.6 % (n=40)	87.7 % (n
	Seizures	15.4 % (n=32)	17.5 % (n=10)	0.00027/4.50	39.0 % (n=16)	32.1 % (n
**Gastrointestinal system**	Gastroesophageal reflux	61.5 % (n=40)	33.3 % (n=19)	0.0022/3.17	58.5 % (n=24)	43.2 % (n
	Protein-losing enteropathy	13.9 % (n=9)	5.3 % (n=3)	NS/2.87	14.6 % (n=6)	7.4 % (n
	Feeding tube	35.4 % (n=23)	8.8 % (n=5)	0.00049/5.62	24.4 % (n=10)	22.2 % (n
	Chronic diarrhea	26.2 % (n=17)	8.8 % (n=5)	0.017/3.65	17.1 % (n=7)	18.5 % (n
**Urinary tract/kidney**	Proteinuria	24.6 % (n=16)	10.5 % (n=6)	NS/2.75	17.1 % (n=7)	18.5 % (n
	Nephrotic syndrome	9.2 % (n=6)	7.0 % (n=4)	NS/1.34	4.9 % (n=2)	9.9 % (n
	Inability to fully empty bladder	4.6 % (n=3)	0 %	NS/NA	2.4 % (n=1)	2.5 % (n
**Other**	Behavior issues	30.8 % (n=20)	29.8 % (n=17)	NS/1.045	31.7 % (n=13)	29.6 % (n
	None of the above	0 %	7.0 % (n=4)	0.045/NA	0 %	4.9 % (n

Table S10 - **Correlation of clinical features with overall phenotypic severity in PMM2-CDG.** Clinical manifestations significantly associated with severe phenotypes are highlighted in green. Statistical significance was calculated with the Fisher exact test and statistical significance was set at *p*-value ≤0.05.

	Other clinical features	Overall phenotypic severity*		
		Severe (n=40)	Moderate (n=82)	*p*-value/OR
**Liver**	Hepatomegaly/hepatosplenomegaly	32.5 % (n=13)	23.2 % (n=19)	NS/1.59
	Elevated liver transaminases	60 % (n=24)	50 % (n=41)	NS/1.50
**Heart**	Pericardial effusion	40 % (n=16)	9.8 % (n=8)	0.00017/6.06
	Cardiomyopathy	12.5 % (n=5)	2.4 % (n=2)	0.038/5.62
**Skin, joints and bone**	Ichthyosis	2.5 % (n=1)	2.4 % (n=2)	NS/1.03
	Arthrosis/arthralgia/joint contractures	7.5 % (n=3)	6.1 % (n=5)	NS/1.25
	Osteopenia/osteoporosis	37.5 % (n=15)	21.9 % (n=18)	NS/2.12
**Blood**	Thrombocytopenia	25 % (n=10)	4.9 % (n=4)	0.0019/6.39
	Delayed wound healing	25 % (n=10)	10.9 % (n=9)	NS/2.68
	Bruising tendency	37.5 % (n=15)	20.7 % (n=17)	NS/2.28
	Persistent anemia	20 % (n=8)	12.2 % (n=10)	NS/1.79
**Brain/Central nervous system**	Intellectual disability	85 % (n=34)	58.5 % (n=48)	0.0039/3.97
	Stroke-like episodes	32.5 % (n=13)	28.1 % (n=23)	NS/1.23
	Microcephaly	15 % (n=6)	10.9 % (n=9)	NS/1.43
	Hypotonia	95 % (n=38)	89.0 % (n=73)	NS/2.33
	Seizures	45 % (n=18)	29.3 % (n=24)	NS/1.97
**Gastrointestinal system**	Gastroesophageal reflux	70 % (n=28)	37.8 % (n=31)	0.0010/3.79
	Protein-losing enteropathy	20 % (n=8)	4.9 % (n=4)	0.019/4.80
	Feeding Tube	45 % (n=18)	12.2 % (n=10)	0.00014/5.79
	Chronic diarrhea	30 % (n=12)	12.2 % (n=10)	0.023/3.05
**Urinary tract/kidney**	Proteinuria	32.5 % (n=13)	10.9 % (n=9)	0.0056/3.86
	Nephrotic syndrome	17.5 % (n=7)	3.7 % (n=3)	0.014/5.50
	Inability to fully empty bladder	5 % (n=2)	1.2 % (n=1)	NS/4.21

**Other**	Behavior issues	35 % (n=14)	28.1 % (n=23)	NS/1.38
	None of the above	0 %	4.9 % (n=4)	NS/0

Legend: NS – Not significant; OR – Odds ratio

*Overall phenotypic severity was assessed by the participant according to immune-related manifestations and all the other clinical manifestations presented by the patient. Participants could classify overall phenotypic severity using a 4-option scale (mild, moderate, severe and very severe)

Table S11 – **Immune-related manifestations and other clinical data of PMM2-CDG patients harboring the Arg141His/R141H variant.** Forty-one PMM2-CDG patients from 16countries harbor the R141H mutation. In total, 19 different genotypes are represented. The most common genotype is the R141H/V231M (n=8/41).

Variant A	Variant B	Age range (current)	Gender	Country (living)	*Immune-related manifestations*				
					Infection profile	Allergy profile	Autoimmunit y profile	Lab alterations	Vaccinatio[illegible] response[illegible]
Arg141His /R141H[α]	Val231Met/ V231M	7-12 mo (1/8)<br>4-10 yrs (3/8)<br>11-20 yrs (1/8)<br>Deceased (3/8):<br>at 6 mo (1/8), at 21 mo (1/8) and at 2 yrs (1/8)	M (5/8)<br>F (3/8)	Argentina (1/8)<br>Australia (1/8)<br>Brazil (1/8)<br>Estonia (1/8)<br>Italy (1/8)<br>UK (1/8)<br>USA (1/8)	Relevant infections (5/8)<br>**Frequent/chronic:**<br>Cold/flu, otitis (2/5); RT, lung, UTI/kidney, blood, skin (1/5)<br>**Treatment ineffectiveness:**<br>IVIG (2/5); antibiotics (1/5)	**Allergies** (3/8)<br>Food (2/3); asthma, rhinitis, sinusitis, dermatitis (1/3)	Glomerulone phritis and celiac disease (1/8)	**Constant:**<br>HGG (1/8)<br>**Sporadic:**<br>High IG, lymphopenia, neutrophilia, basophilia, eosinophilia (1/8)<br>**Present when younger:**<br>HGG, high IG (1/8)<br>**Only during infections:**<br>Leukocytosis (3/8); neutrophilia, monocytosis (1/8)	Never vaccin[illegible] due to HCP ac[illegible] (1/8)<br>Not taken a[illegible] vaccines (2/[illegible]<br>**Vaccinatio[illegible] ineffectivene[illegible]**<br>Failure to generate/los[illegible] protective antibodies ([illegible]<br>**Relevant AD[illegible]**<br>Seizures (1/[illegible]

Variant A	Variant B	Age range (current)	Gender	Country (living)	*Immune-related manifestations*				
					Infection profile	Allergy profile	Autoimmunit y profile	Lab alterations	Vaccinatio response
	Pro113Leu/ P113L*	13 mo-3 yrs (1/6) 4-10 yrs (1/6) +20yrs (4/6)	M (5/6) F (1/6)	Israel (2/6) Netherlan ds (1/6) USA (3/6)	Relevant infections (2/6) **Frequent/chronic:** RT (2/2); GI (1/2)	**Allergies** (3/6) Asthma (2/6); sinusitis, animal, hives, eczema (1/6)	NR	**Sporadic:** Basophilia (2/6)	Not taken a vaccines (2/ Patient beca extremely up (1/6)
	Phe119Leu/ F119L	13 mo-3 yrs (1/4) 4-10 yrs (2/4) 11-20 yrs (1/4)	M (2/4) F (2/4)	France (1/4) Netherlan ds (2/4) USA (1/4)	Relevant infections (4/4) **Frequent/chronic:** Cold/flu, otitis, RT, GI (2/4) Lung, UTI/kidney (1/4) **Severe:** Blood (1/4) **Treatment ineffectiveness:** Antibiotics (1/4)	**Allergies** (1/4) Drug, Hives	NR	**Only during infections:** HGG, Leukocytosis (1/4)	**Vaccinatio ineffective** (3 Failure to generate/los protective antibodies (1 Later the pat developed a infection by agent he has b vaccinated (2 **Relevant AI** Generalize infection (1/

Variant A	Variant B	Age range (current)	Gender	Country (living)	*Immune-related manifestations*				
					Infection profile	Allergy profile	Autoimmunit y profile	Lab alterations	Vaccinatio response
Arg141His /R141H[α]	Ala108Val/ A108V	13 mo-3 yrs (1/3) 4-10 yrs (1/3) +20 yrs (1/3)	M (1/3) F (2/3)	Australia (1/3) France (1/3) Italy (1/3)	Relevant infections (2/3) **Frequent/chronic:** Cold/flu, RT (2/2); titis, tooth/mouth (1/2) **Severe:** Heart (1/2) **Treatment ineffectiveness:** Immune modulators (1/3)	**Allergies** (1/3) Rhinitis	NR	**Constant:** Lymphocytosis (1/3) **Only during infections:** Leukocytosis, neutrophilia (1/3)	Vaccinated (3 No relevant A
	Asn216Ile/ N216I	4-10 yrs (1/2) 11–20 yrs (1/2)	M (2/2)	Italy (1/2) USA (1/2)	Relevant infections (1/2) **Frequent/chronic:** Cold/flu	**Allergies** (1/2) Food and eczema	NR	**Sporadic:** Leukopenia (1/2) **When younger:** High IGs, leukocytosis (1/2)	Vaccinated (2 No relevant A
	Pro69Ser/ P69S	13 mo-3 yrs (1/2) +20 yrs (1/2)	M (1/2) F (1/2)	Australia (1/2) Canada (1/2)	NR	NR	NR	NR	Vaccinated (2 No relevant A

Variant A	Variant B	Age range (current)	Gender	Country (living)	*Immune-related manifestations*				
					Infection profile	Allergy profile	Autoimmunit y profile	Lab alterations	Vaccinatio response
	Glu139Lys/ E139K	4-10 yrs (1/2) 11-20 yrs (1/2)	F (2/2)	France (1/2) USA (1/2)	NR	NR	NR	**Constant:** Basophilia (1/2) **Only during infections:** High IGs	Vaccinated (2 **Vaccinatio ineffectivene** Later the pat developed a infection by agent he has b vaccinated (1 No relevant A
	Thr237Met/ T237M	11-20 yrs (1/2) +20yrs (1/2)	M (1/2) F (1/2)	Spain (2/2)	Relevant infections (1/2)[µ]	NR	NR	**Sporadic:** Eosinophilia (1/2)	Vaccinated (2 No relevant A
Arg141His /R141H[α]	Phe183Ser/F 183S	4-10 yrs (2/2)	M (1/2) F (1/2)	Italy (1/2) Russia (1/2)	Relevant infections (1/2) **Frequent/chronic:** Cold/flu, RT, lung, GI **Treatment ineffectiveness:** Immune modulators	**Allergies** (1/2) Drug and hives	Inflammatory bowel disease (1/2)	**Constant:** Leukocytosis (1/2) **Only during infections:** Neutropenia, basophilia, monocytosis, eosinophilopenia	Never vaccin as HCP nev recommende (1/2)
	Ile132Thr/ I132T	4-10 yrs	M (1/1)	UK	Relevant infections[γ]	NR	NR	NR	Vaccinated No relevant A

Variant A	Variant B	Age range (current)	Gender	Country (living)	*Immune-related manifestations*				
					Infection profile	Allergy profile	Autoimmunit y profile	Lab alterations	Vaccinatio response
	Tyr76Cys/ Y76C	4-10 yrs	F (1/1)	Finland	Relevant infections[μ] **Treatment ineffectiveness:** NR	Animal, food, and eczema	NR	NR	Vaccinated No relevant A
	Gly214Ser/G 214S	>20 yrs	F (1/1)	Slovenia	NR	NR	NR	NR	Vaccinated No relevant A
	Gly208Ala/ G208A	4-10 yrs	F (1/1)	USA	Relevant infections **Frequent/chronic:** Cold/flu, GI, UTI/kidney **Treatment ineffectiveness:** Antibiotics	NR	NR	NR	Vaccinated No relevant A
Arg141His /R141H[α]	Val129Met/ V129M	13 mo-3 yrs	M (1/1)	Australia	NR	NR	NR	**Constant:** Leukocytosis **Sporadic:** DGG, lymphopenia, monocytosis **Only during infections:** HGG, leukocytosis, lymphocytosis, neutrophilia, basophilia	Vaccinated No relevant A

New insights into immunological involvement in Congenital Disorders of Glycosylation (CDG) from a people-centric approach

Supplementary Material

Variant A	Variant B	Age range (current)	Gender	Country (living)	*Immune-related manifestations*				
					Infection profile	Allergy profile	Autoimmunit y profile	Lab alterations	Vaccinatio[illegible] response[illegible]
	Leu32Arg/ L32R	11-20 yrs	M (1/1)	Spain	NR	Food, dermatitis and trees/pollen	NR	**Constant:** Eosinophilia	Vaccinate[illegible] No relevant [illegible]
	Tyr64Cys/ Y64C	4-10 yrs	M (1/1)	Spain	Relevant infections **Frequent/chronic:** Otitis, RT, GI	NR	NR	**Present when younger:** DGG, lymphopenia	**Vaccinatio[illegible] ineffectiv[illegible]** Failure to produce/los[illegible] protectiv[illegible] antibodie[illegible]
	Asp223Asn/ D223N	0-6 mo	M (1/1)	Italy	NR	NR	NR	**Only during infections:** Leukopenia, neutropenia	Vaccinate[illegible] No relevant [illegible]
	Cys9Tyr/ C9Y	4-10 yrs	F (1/1)	France	Relevant infections **Frequent/chronic:** RT, GI, skin	NR	NR	**Constant:** DGG, basophilopenia	Vaccinate[illegible] No relevant [illegible]

Variant A	Variant B	Age range (current)	Gender	Country (living)	*Immune-related manifestations*				
					Infection profile	Allergy profile	Autoimmunit y profile	Lab alterations	Vaccinatio response
Arg141His /R141H[α]	Val43Met/V 43M	4-10 yrs	F (1/1)	UAE	Relevant infections[μ]	Food, dermatitis and eczema	NR	NR	Vaccinated No relevant A
**Total**					56.10% (n=23/41) with relevant infections	31.71% (n=13/41) with allergies	4.88% (n=2/41) with autoimmune diseases		Relevant ADR % (2/41) Vaccinatio ineffectivene 14.6 % (n=6/

Legend: ADR – Adverse reactions; ART – Arthrosis, arthralgia and joint contractures; BI - Behaviour issues; BT – Bruising tendency; CD - Chronic diarrhea; CM – Cardiomyopathy; DWH - Delayed wound healing; F – Female; FTube – Feeding tube; GI – Gastrointestinal; GR – Gastroesophageal reflux; HEPM/HEPSPL – Hepatomegaly/hepatosplenomegaly; HYP – Hypotonia; ICT – Ichthyosis; ID - Intellectual disability; IVIG – Intravenous immunoglobulins; LAIR – Lung and airways; M - Male; MC – Microcephaly; mo – Months; NR – Not reported; NS – Nephrotic syndrome; OP – Osteopenia/osteoporosis; PA – Persistent anemia; PE – Pericardial effusion; PLE – Protein-losing enteropathy; PN – Proteinuria; RT – Respiratory tract; SLE - Stroke-like episodes; SZ – Seizures; TP - Thrombocytopenia; TRANS – Transaminases; UTI – Urinary tract infections; yrs – Years

Only generalized infection, seizures and/or repetitive and/or severe allergic episodes were considered clinically relevant vaccination-related ADR.

*Among these patients, there was one that instead of the R141H variant has the R114H variant, which is a shorter, but equivalent isoform.
[α]PN significantly correlated with the R141H mutation (p= 0.027/OR= 2.91).
[β]A patient reported a negative response to pertussis vaccination (patient became very unsettled, upset, had fever and the episode prolonged itself in time) following this HCP recommended vaccination suspension. The patient's brother – also PMM2-CDG – was also advised not to take all vaccines due to brother's previous experience.
[μ]The patient was reported to have only infections triggering other clinical manifestations.
[γ] The patient was reported to have only severe infections.

Table S12 - **Immune-related manifestations and other clinical data of PMM2-CDG patients not harboring the Arg141His/R141H variant**. Twenty-five PMM2-CDG patients from 9 countries (predominance of southern European countries). In total, 21 different genotypes are represented. The most common variant is the Arg123Gln/R123Q (n=6/25).

Variant A	Variant B	Age range (current)	Gender	Country (living)	*Immune-related manifestations*				
					Infection profile	Allergy profile	Autoimmunity profile	Lab alterations	Vaccin[illegible] respo[illegible]
Arg123Gln/R123Q	Thr226Ser/ T226S	+20 yrs (2/2)	M (1/2) F (1/2)	Spain (2/2)	NR	**Allergies** (2/2) Asthma, animal, dermatitis (2/2) ; pollen (1/2)	NR	NR	Vaccinat[illegible] No rel[illegible] AD[illegible]
	Val231Met /V231M	13 mo-3 yrs (1/2) 11-20 yrs (1/2)	M (1/2) F (1/2)	Italy (1/2) Estonia (1/2)	Relevant infections (2/2) **Frequent/chronic:** Cold/flu, RT, lung, heart, GI (1/2) **Severe:** Blood (1/2) **Treatment ineffectiveness:** IVIG and immune modulators (1/2)	NR	NR	**Constant:** Leukopenia (1/2) **Only during infections:** DGG, Lymphocytosis (1/2)	Vaccinat[illegible] No rel[illegible] AD[illegible]

Variant A	Variant B	Age range (current)	Gender	Country (living)	*Immune-related manifestations*				
					Infection profile	Allergy profile	Autoimmunity profile	Lab alterations	Vaccin respo
	Ala108Val /A108V	4-10 yrs (1/1)	M (1/1)	Ireland	NR	Animal	NR	NR	Vaccir No rel AD
	Gly214Ser /G214S	4-10 yrs (1/1)	M (1/1)	Italy	NR	Rhinitis	NR	NR	Vaccir No rel AD
Pro113Leu /P113L	Thr237Arg/ T237R	4-10 yrs (2/2)	F (2/2)	France (1/2) USA (1/2)	Relevant infections (2/2) **Frequent/chronic:** Cold/flu, otitis, RT (1/2)	**Allergies** (2/2) Rhinitis, drugs (1/2)	NR	**Constant:** Leukocytosis, lymphopenia, neutropenia, basophilopenia, eosinophilopenia (1/2) **Sporadic:** Monocytosis, monocytopenia (1/2) **Only during infections:** High IGs	**Vaccin ineffecti** Failu generate protec antibodi No rel ADR

Variant A	Variant B	Age range (current)	Gender	Country (living)	*Immune-related manifestations*				
					Infection profile	Allergy profile	Autoimmunity profile	Lab alterations	Vaccin respo
	Phe207Ser /F207S	4-10 yrs (1/1)	F (1/1)	Spain	Relevant infections **Frequent/chronic:** Otitis	NR	NR	**Sporadic:** Monocytosis, eosinophilia	**Relevan** Genera infectio SL
	Pro113Leu /P113L	4-10 yrs (1/1)	F (1/1)	Spain	NR	Suture thread	NR	NR	Vaccin No rel AD
	Arg123Ter /R123*	4-10 yrs (1/1)	F (1/1)	Chile	NR	NR	NR	**Constant:** NR **Sporadic:** Basophilia **When younger:** NR **Only during infections:** Neutropenia	Vaccin No rel AD
Ala108Val /A108V	Phe157Ser /F157S	4-10 yrs (1/1)	M (1/1)	Australia	NR	Sinusitis	NR	**Sporadic:** Basophilia **Only during infections:** Leukocytosis	Vaccin No rel AD
	T171Nfs*11 (511dupA)	4-10 yrs (1/1)	F (1/1)	Italy	NR	NR	NR	NR	Vaccin No rel AD

Variant A	Variant B	Age range (current)	Gender	Country (living)	***Immune-related manifestations***				
					Infection profile	Allergy profile	Autoimmunity profile	Lab alterations	Vaccin resp
Thr237Met/T237M	Phe157Ser /F157S	13 mo-3 yrs (1/2) 4-10 yrs (1/2)	M (1/ 2) F (1/2)	Portugal (1/2) Spain (1/2)	Relevant infections (1/2) **Frequent/chronic:** Tooth/mouth	**Allergies** (1/2) Food, dermatitis	NR	**Sporadic:** DGG, high IGs, leukopenia, leukocytosis, lymphopenia, neutrophilia, monocytosis, eosinophilia, eosinophilopenia (1/2)	Vaccin No rel AD
	c.640-9T>G (IVS7-9T)	11-20 yrs (1/1)	F (1/1)	Spain	NR	NR	NR	**Sporadic:** Eosinophilopenia	Vaccin No rel AD
Val231Met /V231M	Asp188Gly /D188G	4-10 yrs (1/1)	F (1/1)	USA	NR	NR	NR	NR	Vaccin No rel AD
Pro69Ser/ P69S	Asp148Asn/D148N	13 mo-3yrs (1/1)	M (1/1)	UK	NR	NR	NR	NR	Vaccin No rel AD
Phe157Ser /F157S	Leu32Arg /L32R	13 mo-3 yrs (1/1)	F (1/1)	Spain	Relevant infections **Frequent/chronic:** Cold/flu, RT, tooth/mouth, lung, GI	NR	NR	**Constant:** DGG, leukocytosis	Vaccin No rel AD

New insights into immunological involvement in Congenital Disorders of Glycosylation (CDG) from a people-centric approach

Supplementary Material

Variant A	Variant B	Age range (current)	Gender	Country (living)	***Immune-related manifestations***				
					Infection profile	Allergy profile	Autoimmunity profile	Lab alterations	Vaccin respo
Glu139Lys /E139K	Phe207Ser /F207S	4-10 yrs (1/1)	M (1/1)	Spain	Relevant infections **Frequent/chronic:** Otitis	NR	NR	NR	Vaccir No rel AD
Ile132Thr / I132T	Phe144Le u / F144L	4-10 yrs (1/1)	F (1/1)	Australia	Relevant infections **Frequent/chronic:** Cold/flu, GI, skin **Treatment ineffectiveness:** Antibiotics	NR	NR	NR	Vaccir No rel AD
Val129Met /V129M	Arg21Gly/ R21G	11-20 yrs	M (1/1)	France	Relevant infections **Frequent/chronic:** Cold/flu, RT,	NR	Rhinitis	NR	Vaccir No rel AD
c.640-9T>G (IVS7-9T)	Asp65Tyr/ D65Y	11-20 yrs	F (1/1)	Spain	NR	NR	NR	NR	Vaccir **Relevan** Allergic
Ser47L/ S47L	Gln33Pro/ Q33P	4-10 yrs	M (1/1)	USA	NR	NR	NR	NR	**Vaccin ineffe** failur

New insights into immunological involvement in Congenital Disorders of Glycosylation (CDG) from a people-centric approach

Supplementary Material

Variant A	Variant B	Age range (current)	Gender	Country (living)	*Immune-related manifestations*				
					Infection profile	Allergy profile	Autoimmunity profile	Lab alterations	Vaccin respo
									generate protec antibo No rele AD
Thr237Arg/ T237R	Arg162Trp/R162W	4-10 yrs	F (1/1)	Portugal	NR	NR	NR	**Sporadic:** Leukocytosis, lymphocytosis, neutrophilia, monocytosis, eosinophilopenia	Vaccin No rele AD
**Total**					40% (n=10/25) with relevant infections	36% (n=9/25) with allergies	0 % with autoimmune diseases		Relevant % (2/ Vaccin ineffectiv % (n=

Legend: ADR – Adverse reactions; ART – Arthrosis, arthralgia and joint contractures; BI - Behaviour issues; BT – Bruising tendency; CD - Chronic diarrhea; CM – Cardiomyopathy; DWH - Delayed wound healing; F – Female; FTube – Feeding tube; GI – Gastrointestinal; GR – Gastroesophageal reflux; HEPM/HEPSPL – Hepatomegaly/hepatosplenomegaly; HYP – Hypotonia; ICT – Ichthyosis; ID -Intellectual disability; IVIG – Intravenous immunoglobulins; LAIR – Lung and airways; M- Male; MC – Microcephaly; mo – Months; NR – Not reported; NS – Nephrotic syndrome; OP – Osteopenia/osteoporosis; PA – Persistent anemia; PE – Pericardial effusion; PLE – Protein-losing enteropathy; PN – Proteinuria; RT – Respiratory tract; SLE - Stroke-like episodes; SZ – Seizures; TP - Thrombocytopenia; TRANS – Transaminases; UTI – Urinary tract infections; yrs – Years

New insights into immunological involvement in Congenital Disorders of Glycosylation (CDG) from a people-centric approach

Supplementary Material

Table S13 – **Analysis of the pathogenicity of *PMM2* variants listed in the ImmunoCDGQ with a set of *in silico* prediction tools**.

**Gene symbol:** *PMM2*		**UNIPROT ID:** O15305		**Chromosomal location:** 16p13.2							
**Transcript Variant**	**Protein Variant**	**Variation type**	**dB SNP**	**Gene Region**	**Translation impact**	**DANN**	**FATHMM-MKL**	**Mutation Taster**	**Provean**	**PolyPhen-2**	**SIFT**
c.422G>A	Arg141His/ R141H	SNV	rs28936415	Exonic	Missense	0.9993	DM (score: 0.973)	DC automatic (P: 0.999)	DL (score: -4.80)	Possibly DM (HumVar score: 0.510)	DM (score: 0.
c.341G>A[#]	Arg114His/ R114H									NA	NA
c.338C>T	Pro113Leu/ P113L	SNV	rs80338700	Exonic	Missense	0.9987	DM (score: 0.9761)	DC automatic (P: 0.999)	DL (score: -9.13)	Probably DM (HumVar score: 0.997)	DM (score: 0.
c.691G>A	Val231Met/ V231M	SNV	rs80338707	Exonic	Missense	0.9979	DM (score: 0.9733)	DC automatic (P: 0.999)	DL (score: -2.88)	Probably DM (HumVar score: 0.991)	DM (score: 0.
c.323C>T	Ala108Val/ A108V	SNV	rs200503569	Exonic	Missense	0.996	DM (score: 0.9295)	DC (P: 0.999)	DL (score: -3.53)	BG (HumVar score: 0.070)	DM (score: 0.
c.368G>A	Arg123Gln/ R123Q	SNV	rs141498002	Exonic	Missense	0.9995	DM (score: 0.9735)	DC automatic (P: 0.999)	DL (score: -3.77)	Possibly DM (HumVar score: 0.844)	DM (score: 0.
c.710C>T	Thr237Met/ T237M	SNV	rs80338708	Exonic	Missense	0.9989	DM (score: 0.9636)	DC automatic (P: 0.999)	DL (score: -5.62)	Probably DM (HumVar score: 1)	DM (score: 0.
c.470T>C	Phe157Ser/ F157S	SNV	rs190521996	Exonic	Missense	0.9988	DM (score: 0.9668)	DC (P: 0.999)	DL (score: -5.65)	Probably DM (HumVar score: 1)	DM (score: 0.

**Gene symbol:** *PMM2*		**UNIPROT ID:** O15305		**Chromosomal location:** 16p13.2							
**Transcript Variant**	**Protein Variant**	**Variation type**	**dB SNP**	**Gene Region**	**Translation impact**	**DANN**	**FATHMM-MKL**	**Mutation Taster**	**Provean**	**PolyPhen-2**	**SIFT**
c.357C>A	Phe119Leu/ F119L	SNV	rs80338701	Exonic	Missense	0.9967	DM (score: 0.9319)	DC automatic (P: 0.999)	DL (score: -5.82)	Probably DM (HumVar score: 0.994)	DM (score: 0.[illegible]
c.205C>T	Pro69Ser/ P69S	SNV	rs769648248	Exonic	Missense	0.3137	DM (score: 0.8629)	DC (P: 0.998)	N (score: 0.54)	BG (HumVar score: 0.329)	T (score: [illegible]
c.710C>G	Thr237Arg/ T237R	SNV	rs80338708	Exonic	Missense	0.9934	DM (score: 0.9699)	DC automatic (P: 0.999)	DL (score: -5.47)	Probably DM (HumVar score: 1)	DM (score: 0.[illegible]
c.415G>A	Glu139Lys/ E139K	SNV	rs80338703	Exonic	Missense	0.9986	DM (score: 0.9315)	DC automatic (P: 0.999)	N (score: -2.38)	BG (HumVar: 0.132)	T (score: 0.[illegible]
c.395T>C	Ile132Thr / I132T	SNV	rs80338702	Exonic	Missense	0.9978	DM (score: 0.9445)	DC (P: 0.999)	DL (score: -4.45)	Probably DM (HumVar score: 0.988)	DM (score: 0.[illegible]
c.647A>T	Asn216Ile / N216I	SNV	rs78290141	Exonic	Missense	0.9897	DM (score: 0.9744)	DC (P: 0.999)	DL (score: -8.64)	Probably DM (HumVar score: 0.998)	DM (score: 0.[illegible]
c.640G>A	Gly214Ser/ G214S	SNV	rs1555453238	Exonic	Missense	0.9982	DM (score: 0.975)	DC (P: 0.999)	DL (score: -5.74)	Probably DM (HumVar score: 0.938)	DM (score: 0.[illegible]
c.548T>C	Phe183Ser/ F183S	SNV	rs780581250	Exonic	Missense	0.9987	DM (score: 0.9546)	DC (P: 0.999)	DL (score: -7.53)	Probably DM (HumVar score: 0.999)	DM (score: 0.[illegible]
c.385G>A	Val129Met/ V129M	SNV	rs104894525	Exonic	Missense	0.999	DM (score: 0.894)	DC (P: 0.999)	DL (score: -2.52)	Probably DM (HumVar score: 0.982)	DM (score: 0.[illegible]

**Gene symbol:** *PMM2*		**UNIPROT ID:** O15305		**Chromosomal location:** 16p13.2							
**Transcript Variant**	**Protein Variant**	**Variation type**	**dB SNP**	**Gene Region**	**Translation impact**	**DANN**	**FATHMM-MKL**	**Mutation Taster**	**Provean**	**PolyPhen-2**	**SIFT**
c.677C>G	Thr226Ser/ T226S	SNV	rs80338706	Exonic	Missense	0.9946	DM (score: 0.9699)	DC automatic (P: 0.999)	DL (score: -3.78)	Probably DM (HumVar score: 0.918)	DM (score: 0.[illegible]
c.620T>C	Phe207Ser/ F207S	SNV	rs532870929	Exonic	Missense	0.9985	DM (score: 0.9563)	DC (P: 0.999)	DL (score: -7.70)	Probably DM (HumVar score: 1)	DM (score: 0.[illegible]
c.95T>G	Leu32Arg/ L32R	SNV	rs104894533	Exonic	Missense	0.9974	DM (score: 0.9761)	DC automatic (P: 0.999)	DL (score: -4.92)	Probably DM (HumVar score: 0.999)	DM (score: 0.[illegible]
c.640-9T>G (IVS7-9T)µ	-	SNV	rs370160676	Intronic	Splicing	0.8494	NA	DC (P:1) due to affected splicing	NA	NA	NA
c.442G>A	Asp148Asn/ D148N	SNV	rs148032587	Exonic	Missense	0.9991	DM (score: 0.9731)	DC automatic (P: 0.999)	DL (score: -4.63)	Probably DM (HumVar score: 0.996)	DM (score: 0.[illegible]
c.563A>G	Asp188Gly/ D188G	SNV	rs80338704	Exonic	Missense	0.9986	DM (score: 0.9686)	DC automatic (P: 0.999)	DL (score: -6.67)	Probably DM (HumVar score: 1)	DM (score: 0.[illegible]
c.227A>G	Tyr76Cys/ Y76C	SNV	rs1440183322	Exonic	Missense	0.9971	DM (score: 0.9495)	DC (P: 0.999)	DL (score: -7.08)	Probably DM (HumVar score: 0.962)	DM (score: 0.[illegible]
c.430T>C	Phe144Leu/ F144L	SNV	rs150719105	Exonic	Missense	0.9976	DM (score: 0.8766)	DC (P: 0.999)	DL (score: -5.72)	Possibly DM (HumVar score: 0.791)	DM (score: 0[illegible]
c.140C>T	Ser47Leu/ S47L	SNV	rs138306798	Exonic	Missense	0.9993	DM (score: 0.9774)	DC (P: 0.999)	DL (score: -5.20)	Probably DM (HumVar score: 0.995)	DM (score: 0.[illegible]

New insights into immunological involvement in Congenital Disorders of Glycosylation (CDG) from a people-centric approach

Supplementary Material

**Gene symbol:** *PMM2*		**UNIPROT ID:** O15305		**Chromosomal location:** 16p13.2							
**Transcript Variant**	**Protein Variant**	**Variation type**	**dB SNP**	**Gene Region**	**Translation impact**	**DANN**	**FATHMM-MKL**	**Mutation Taster**	**Provean**	**PolyPhen-2**	**SIFT**
c.98A>C	Gln33Pro/ Q33P	SNV	rs773229229	Exonic	Missense	0.9933	DM (score: 0.9095)	DC (P: 0.999)	DL (score: -2.95)	Probably DM (HumVar score: 0.915)	T (score: 0.
c.127G>A	Val43Met/ V43M	SNV	rs376754460	Exonic	Missense	0.9991	DM (score: 0.9211)	DC (P: 0.999)	N (score: -0.98)	Probably DM (HumVar score: 0.982)	DM (score: 0.
c.623G>C	Gly208Ala/ G208A	SNV	rs398123309	Exonic	Missense	0.9976	DM (score: 0.9754)	DC automatic (P: 0.999)	DL (score: -5.78)	Probably DM (HumVar score: 0.987)	DM (score: 0.
c.367C>T[β]	Arg123Ter/ R123*	SNV	rs191295403	Exonic	Nonsense	0.9966	DM (score: 0.9042)	DC automatic (P:1)	NA	NA	DM due to (score: N
c.193G>T	Asp65Tyr/ D65Y	SNV	rs104894527	Exonic	Missense	0.9948	DM (score: 0.9669)	DC automatic (P: 0.999)	DL (score: -7.28)	Possibly DM (HumVar score: 0.873)	DM (score: 0.
c.191A>G	Tyr64Cys/ Y64C	SNV	NA	Exonic	Missense	0.9977	DM (score: 0.9257)	DC (P: 0.994)	DL (score: -6.06)	Probably DM (HumVar score: 0.996)	DM (score: 0.
c.484C>T	Arg162Trp/ R162W	SNV	rs104894526	Exonic	Missense	0.9797	DM (score: 0.8553)	DC (P: 0.998)	DL (score: -5.42)	BG (HumVar score: 0.017)	DM (score: 0.
c.667G>A	Asp223Asn/ D223N	SNV	rs201960869	Exonic	Missense	0.9993	DM (score: 0.9736)	DC (P: 0.999)	DL (score: -3.53)	Probably DM (HumVar score: 0.991)	DM (score: 0.
c.26G>A	Cys9Tyr/C9Y	SNV	rs104894532	Exonic	Missense	0.9916	DM (score: 0.9417)	DC (P: 0.999)	DL (score: -5.49)	Possibly DM (HumVar: 0.664)	DM (score: 0.

**Gene symbol:** *PMM2*		**UNIPROT ID:** O15305		**Chromosomal location:** 16p13.2							
**Transcript Variant**	**Protein Variant**	**Variation type**	**dB SNP**	**Gene Region**	**Translation impact**	**DANN**	**FATHMM-MKL**	**Mutation Taster**	**Provean**	**PolyPhen-2**	**SIFT**
c.61C>G	Arg21Gly/ R21G	SNV	rs758340382	Exonic	Missense	0.9982	DM (score: 0.8014)	DC (P: 0.999)	DL (score: -5.51)	Probabaly DM (HumVar score: 0.938)	DM (score:0.0[illegible]
c.511dupA	Thr171Asnfs Ter11/ T171Nfs*11	INDEL	rs1057516323	Exonic	Frameshift	NA	NA	DC (P:1)	NA	NA	NA

Legend: BG – Benign; DC - Disease-causing; DL – Deleterious; DM- Damaging; INDEL – Insertions and deletions; N - Neutral; NA – Not available; P - Probability; SNV – Single nucleotide variants; T – Tolerated

*Based on ClinVar classification terms and on the work recently published by Sun and Yu, we established an overall mutation pathogenicity cataloguing with each mutation being assigned to one of the following 3 categories ([2]):

1) Pathogenic – when all applied tools predicted the variant as pathogenic and there was complete absence of possible benign effects;
2) Benign – when all applied tools predicted the variant as benign and there was complete absence of possible damaging effects;
3) Both – when predictions of both pathogenic and benign consequences for a variant were made by different tools.

#This is the same variant but originates from transcript (ENST00000566983.1) that encodes a shorter protein isoform with 219 amino acids.

αPredicted by SIFT Indel to cause non-mediated decay of the mRNA and both SIFT Indel and VEST Indel predicted the frameshift effect of this mutation.

µExperimental evidence shows that this mutation led to the activation of a cryptic intronic splice-site ([15]).

βResults in nonsense mRNA mediated decay.

[1] D. Quang, Y. Chen, X. Xie, DANN : a deep learning approach for annotating the pathogenicity of genetic variants, Bioinformatics. 31 (2015) 761–763. doi:10.1093/bioinformatics/btu703.

[2] H. Sun, G. Yu, New insights into the pathogenicity of non-synonymous variants through multi-level analysis, Sci. Rep. 9 (2019). doi:10.1038/s41598-018-38189-9.

[3] H.A. Shihab, M.F. Rogers, J. Gough, M. Mort, D.N. Cooper, I.N.M. Day, T.R. Gaunt, C. Campbell, An integrative approach to predicting the functional effects of non-coding and coding sequence variation, Bioinformatics. 31 (2015) 1536–1543.

doi:10.1093/bioinformatics/btv009.

[4] X. Liu, C. Wu, C. Li, E. Boerwinkle, dbNSFP v3 . 0 : A One-Stop Database of Functional Predictions and Annotations for Human Nonsynonymous and Splice-Site SNVs, Hum. Mutat. 37 (2016) 235–241. doi:10.1002/humu.22932.

[5] H. Venselaar, T.A. te Beek, R.K.P. Kuipers, M.L. Hekkelman, G. Vriend, Protein structure analysis of mutations causing inheritable diseases . An e-Science approach with life scientist friendly interfaces, BMC Bioinformatics. 11 (2010).

[6] J.M. Schwarz, D.N. Cooper, M. Schuelke, D. Seelow, MutationTaster2 : mutation prediction for the deep-sequencing age, Nat. Publ. Gr. 11 (2014) 361–362. doi:10.1038/nmeth.2890.

[7] Y. Choi, G.E. Sims, S. Murphy, J.R. Miller, A.P. Chan, Predicting the Functional Effect of Amino Acid Substitutions and Indels, PLoS One. 7 (2012) e46688. doi:10.1371/journal.pone.0046688.

[8] Y. Choi, A.P. Chan, PROVEAN web server : a tool to predict the functional effect of amino acid substitutions and indels, Bioinformatics. 31 (2015) 2745–2747. doi:10.1093/bioinformatics/btv195.

[9] I.A. Adzhubei, S. Schmidt, L. Peshkin, V.E. Ramensky, A. Gerasimova, P. Bork, A.S. Kondrashov, S.R. Sunyaev, A method and server for predicting damaging missense mutations, Nat. Publ. Gr. 7 (2010) 248–249. doi:10.1038/nmeth0410-248.

[10] N.-L. Sim, P. Kumar, J. Hu, S. Henikoff, G. Schneider, P.C. Ng, SIFT web server : predicting effects of amino acid substitutions on proteins, Nucleic Acids Res. 40 (2012) 452–457. doi:10.1093/nar/gks539.

[11] J. Hu, P.C. Ng, Predicting the effects of frameshifting indels, Genome Biol. 12 (2012).

[12] J. Hu, P.C. Ng, SIFT Indel : Predictions for the Functional Effects of Amino Acid Insertions / Deletions in Proteins, PLoS One. 8 (2013) 1–8. doi:10.1371/journal.pone.0077940.

[13] C. Douville, D.L. Masica, P.D. Stenson, D.N. Cooper, D.M. Gygax, R. Kim, M. Ryan, R. Karchin, Assessing the Pathogenicity of Insertion and Deletion Variants with the Variant Effect Scoring Tool (VEST-Indel), Hum. Mutat. 37 (2016) 28–35. doi:10.1002/humu.22911.

[14] F.-O. Desmet, D. Hamroun, M. Lalande, G. Collod-Béroud, M. Claustres, C. Béroud, Human Splicing Finder : an online bioinformatics tool to predict splicing signals, Nucleic Acids Res. 37 (2009) 1–14. doi:10.1093/nar/gkp215.

[15] A.I. Vega, C. Perez-Cerda, L.R. Desviat, G. Matthijs, M. Ugarte, B. Perez, Functional analysis of three splicing mutations identified in the PMM2 gene: toward a new therapy for congenital disorder of glycosylation type Ia., Hum. Mutat. 30 (2009) 795–803. doi:10.1002/humu.20960.

Table S14 – **Comparative analysis of the effects of wild-type vs mutated residues on PMM2 physico-chemical and structural properties and overall predicted impact on the function/activity of the protein.**

**Mutation**		**Amino acid properties**						**Structure**	
		**Charge/Hydrophobicity**		**Size**		**Conservation**		**Bonds/contacts/conformation**	
**Transcript Variant**	**Protein Variant**	**WT residue**	**MuT residue**	**WT residue**	**MuT residue**	**WT residue**	**MuT residue**	**WT residue**	**MuT resi**
c.422G>A	Arg141His/R141H	Positive (Basic)	Neutral	-	Smaller than WT residue	Highly conserved	-	Forms a H bond with Q at position 177.	Cannot form the same WT resid
c.341G>A	Arg114His/R114H #	NA							
c.338C>T	Pro113Leu/P113L	Neutral (Hydrophobic)		-	Bigger than the WT residue	Highly conserved	-	Located on the surface of the protein. It induces a special backbone conformation due to its rigidity.	Does not have the sam WT resid
c.691G>A	Val231Met/V231M	Neutral (Hydrophobic)		-	Bigger than the WT residue	Very conserved	-	Predicted to be preferentially located on a β-strand, buried in the core of the protein.	Prefers to be in anot structure, as it is too same locat
c.323C>T	Ala108Val/A108V	Neutral (Hydrophobic)		-	Bigger than the WT residue	Very conserved	-	Located in a α-helix on the surface of the enzyme.	Does not prefer α- secondary r
c.368G>A	Arg123Gln/R123Q	Positive (Basic)	Neutral (Polar)	-	Smaller than the WT residue	Highly conserved	-	Forms a H bond and salt bridge with E at position 121 and a salt bridge D at position 181.	Cannot form the same bridges as the W

Mutation		Amino acid properties						Structure	
		Charge/Hydrophobicity		Size		Conservation		Bonds/contacts/conformation	
**Transcript Variant**	**Protein Variant**	**WT residue**	**MuT residue**	**WT residue**	**MuT residue**	**WT residue**	**MuT residue**	**WT residue**	**MuT resi**
c.710C>T	Thr237Met/T237M	Neutral (Polar)	Neutral More hydrophobic than the WT	-	Bigger than the WT residue	Highly conserved	-	Located in the core of the protein. Forms H bonds with T at position 16 and A at position 233.	MuT increased size car conformation. It canno H bonds as the W
c.470T>C	Phe157Ser/F157S	More hydrophobic than the MuT residue	Neutral (Polar)	-	Smaller than the WT residue	Conserved	-	Located in the core of the protein.	Smaller residue size empty space in the cor
c.357C>A	Phe119Leu/F119L	Hydrophobic		-	Smaller than the WT residue	Highly conserved	-	Predicted to be preferentially located on a β-strand.	Prefers to be in anot structur
c.205C>T	Pro69Ser/ P69S	More hydrophobic than the MuT residue	Neutral (Polar)	-	Smaller than the WT residue	Not conserved	-	Located in the core of the protein. High rigidity conferring a special local backbone conformation to the protein.	Smaller residue size empty space in the cor
c.710C>G	Thr237Arg/T237R	Neutral (polar) More hydrophobic than the MuT residue	Positive (basic)	-	Bigger than the WT residue	Highly conserved	-	Located in the core of the protein. Forms a H bond with a T at position 16 and A at position 233.	MuT increased size c conformation. It canno H bond as the W

Mutation		Amino acid properties						Structure	
		Charge/Hydrophobicity		Size		Conservation		Bonds/contacts/conformation	
Transcript Variant	Protein Variant	WT residue	MuT residue	WT residue	MuT residue	WT residue	MuT residue	WT residue	MuT resi
c.415G>A	Glu139Lys/E139K	Negative (acidic)	Positive (basic)	-	Bigger than the WT residue	Very conserved	-	Located on the surface of the enzyme. Forms a salt bridge with a K at position 114.	Cannot form the salt b residue
c.395T>C	Ile132Thr/I132T	More hydrophobic than the MuT residue	Neutral (polar)	-	Smaller than the WT residue	Very conserved	-	Located in the core of the protein.	Smaller residue size empty space in the cor
c.647A>T	Asn216Ile/N216I	Neutral (polar)	More hydrophobic than the WT residue	-	Smaller than the WT residue	Highly conserved	-	Predicted to be preferentially located on a turn of the enzyme.	Prefers to be in anot structur
c.640G>A	Gly214Ser/G214S	Hydrophobic	Neutral (polar)	-	Bigger than the WT residue	Highly conserved	-	Located on the surface of the protein. The most flexible of all residues. It can form torsion angles.	Incapable of producin
c.548T>C	Phe183Ser/F183S	More hydrophobic than the MuT residue	Neutral (polar)	-	Smaller than the WT residue	Very conserved	-	Located in the core of the protein. Predicted to be preferentially located on a β-strand.	Smaller residue size empty space in the cor Prefers to be in anot structur
c.385G>A	Val129Met/V129M	Hydrophobic	Hydrophobic	-	Bigger than the WT residue	Conserved	-	Located in the core of the protein. Predicted to be preferentially located on a β-strand.	MuT increased size impact stability. Pr another secondary
c.677C>G	Thr226Ser/T226S	Neutral (polar)	Neutral (Polar)	-	Smaller than the WT residue	Very conserved	-	Located in the core of the protein. Forms a H bond with I at position 220 and D at position 223. Predicted	Smaller residue size t the same bonds as the can also create an em

Mutation		Amino acid properties						Structure	
		Charge/Hydrophobicity		Size		Conservation		Bonds/contacts/conformation	
Transcript Variant	Protein Variant	WT residue	MuT residue	WT residue	MuT residue	WT residue	MuT residue	WT residue	MuT resi
								to be preferentially located on a β-strand.	core of the protein. P another secondary
c.620T>C	Phe207Ser/F207S	More hydrophobic than the MuT residue	Neutral (polar)	-	Smaller than the WT residue	Highly conserved	-	Located in the core of the protein. Predicted to be preferentially located on a β-strand.	Smaller residue size empty space in the cor Prefers to be in anot structur
c.95T>G	Leu32Arg/L32R	Neutral More hydrophobic than the MuT residue	Positive (acidic)	-	Bigger than the WT residue	Conserved	-	Located in a α-helix in the core of the protein.	Prefers to be in anot structur
c.640-9T>G	NA	NA	NA	NA	NA	NA	NA	NA	NA
c.442G>A	Asp148Asn/D148N	Negative (basic)	Neutral (polar)	Similar size	Similar size	Highly conserved	-	Forms a H bond and a salt bridge with an R at position 154.	Cannot form the sar interactio
c.563A>G	Asp188Gly/D188G	Negative (basic)	Neutral (polar) More hydrophobic than the WT residue	-	Smaller than the WT residue	Highly conserved	-	This is a core rigid residue, which forms a H bond and a salt bridge with a R at position 190.	The MuT is more flexi and cannot form the s interactio
c.227A>G	Tyr76Cys/Y76C	Neutral (polar)	Neutal (polar) More hydrophobi	-	Smaller than the WT residue	Conserved	-	Located in the core of the protein. Predicted to be preferentially located on a β-strand.	Smaller residue siz differences can create in the core of the prote in another seconda

Mutation		Amino acid properties						Structure	
		Charge/Hydrophobicity		Size		Conservation		Bonds/contacts/conformation	
Transcript Variant	Protein Variant	WT residue	MuT residue	WT residue	MuT residue	WT residue	MuT residue	WT residue	MuT resi
			c than the WT residue						
c.430T>C	Phe144Leu/F144L	Hydrophob ic	Hydrophob ic	-	Smaller than the WT residue	Very conserved	-	Located in the core of the protein.	Smaller residue siz differences can create in the core of th
c.140C >T	Ser47Leu/S47L	Neutral (polar)	More hydrophobi c than the WT residue	-	Bigger than the WT residue	Very conserved	-	Located on the surface of the protein. Predicted to be preferentially located on a turn of the enzyme.	MuT increased size car conformation. Prefers secondary str
c.98A>C	Gln33Pro/Q33P	Similar properties	Similar properties More hydrophobi c than the WT residue	-	Smaller than the WT residue	Conserved	-	Located in a α-helix.	When a proline is not l the first 3 positions of secondary structure is to H bond
c.127G>A	Val43Met/V43M	Hydrophob ic	Hydrophob ic	-	Bigger than the WT residue	Conserved	-	Located in the core of the protein. Predicted to be preferentially located on a β-strand.	MuT increased size c impact structure and s to be in another secor
c.623G>C	Gly208Ala/G208A	Similar properties, but the MuT is more hydrophobic than the WT residue		-	Bigger than the WT residue	Highly conserved	-	Located in the core of the protein. The most flexible of all residues. It can form torsion angles.	MuT increased size c impact structure a Incapable of producin
c.367C>T	Arg123Ter/R123*	NA							

New insights into immunological involvement in Congenital Disorders of Glycosylation (CDG) from a people-centric approach

Supplementary Material

Mutation		Amino acid properties						Structure	
		Charge/Hydrophobicity		Size		Conservation		Bonds/contacts/conformation	
Transcript Variant	Protein Variant	WT residue	MuT residue	WT residue	MuT residue	WT residue	MuT residue	WT residue	MuT resi
c.193G>T	Asp65Tyr/D65Y	Negative (acidic)	Neutral (polar) More hydrophobi c than the WT residue	-	Bigger than the WT residue	Very conserved	-	Located in the core of the protein. Forms H bonds and salt bridges with Rat position 36 and K at position 40. Also forms H bond with I at position 41.	Cannot form the sar interactions as the Increased size can d conformat
c.191A>G	Tyr64Cys/Y64C	Neutral (polar)	Neutral (polar) More hydrophobi c than the WT residue	-	Smaller than the WT residue	Not conserved	-	Located in the core of the protein.	Smaller residue size empty space in the cor
c.484C>T	Arg162Trp/R162 W	Positive (basic)	Neutral (amphipati c) More hydrophobi c than the WT residue	-	Bigger than the WT residue	Not conserved	-	Located on the surface of the protein.	MuT increased size car conformat
c.667G>A	Asp223Asn/D223 N	Negative (Acidic)	Neutral (Polar)	Similar size	Similar size	Conserved	-	Forms a H bond and salt bridge with a R at position 225 and a salt bridge with a R at position 190.	Cannot form the sar interactions as the
c.26G>A	Cys9Tyr/C9Y	Neural (polar) More hydrophobi c than the	Neutral (polar)	-	Bigger than the WT residue	Conserved	-	Located in the core of the protein.	MuT increased size car conformat

Mutation		Amino acid properties						Structure	
		Charge/Hydrophobicity		Size		Conservation		Bonds/contacts/conformation	
Transcript Variant	Protein Variant	WT residue	MuT residue	WT residue	MuT residue	WT residue	MuT residue	WT residue	MuT resi
		MuT residue							
c.61C>G	Arg21Gly/R21G	Positive (basic)	Neutral (polar) More hydrophobic than the WT residue	-	Smaller than the WT residue	Very conserved	-	Forms a salt bridge with a D at position 14.	Cannot form the same residue. Extra flexibili by the MuT r
c.511dupA	Thr171AsnfsTer11 / T171Nfs*11	NA	NA	NA	NA	NA	NA	NA	NA

Legend: - Not-applicable data; BG – Benign; DM – Damaging; MuT – Mutated; NA – Not available; WT – Wild-type

[#]This is a shorter transcript (ENST00000566983.1), encoding a shorter protein isoform with 220 amino acids

Table S15 - **Laboratory alterations found among control and PMM2-CDG participants.** Reported cellular and antibody altered counts, as well as the timing and/persistence of the anomalies are shown. Only 293 control and PMM2-CDG participants answered this question.

	Control participants					[illegible]	
Frequency / Altered parameter	Constant	Sporadic/last blood tests	When the patient was younger	Only during infections	Never/unknown	Constant	Spor[illegible] blo[illegible]
**Decreased counts**							
Hypogammaglobulinemia	0.3 % (n=1/293)	0.3 % (n=1/293)	0.7 % (n=2/293)	2.4 % (n=7/293)	96.2 % (n=282/293)	2.5 % (n=3/120)	0 %
Dysgammaglobulinemia	0 %	0 %	0.7 % (n=2/293)	2.7 % (n=8/293)	96.9 % (n=284/293)	2.5 % (n=3/120)	2.5 %[#] [illegible]
Leukopenia	0.3 % (n=1/293)	0.7 % (n=2/293)	0.3 % (n=1/293)	1.0 % (n=3/293)	97.6 % (n=286/293)	0.8 %[†] (n=1/120)	2.5% (n[illegible]
Lymphopenia	0.3 % (n=1/293)	0 %	0 %	1.4 % (n=4/293)	98.3 % (n=288/293)	1.7 %[β] (n=2/120)	3.3 % ([illegible]
Neutropenia	0.7 % (n=2/293)	0 %	0.3 % (n=1/293)	1.0 % (n=3/293)	97.9 % (n=287/293)	0.8 % (n=1/120)	0 %
Basophilopenia	0.7 % (n=2/293)	0.3 % (n=1/293)	0 %	0.3 % (n=1/293)	98.6 % (n=289/293)	1.7 % (n=2/120)	0 %
Monocytopenia	0.3 % (n=1/293)	0 %	0.3 % (n=1/293)	0.3 % (n=1/293)	98.9 % (n=290/293)	0 %	0.8 % ([illegible]
Eosinophilopenia	0.7 % (n=2/293)	0.7 % (n=2/293)	0 %	0.3 % (n=1/293)	98.3 % (n=288/293)	0.8 % (n=1/120)	2.5 % ([illegible]
**Increased counts**							
High levels of one or more IGs	1.7 % (n=5/293)	1.4 % (n=4/293)	0.7 % (n=2/293)	3.1 % (n=9/293)	92.8 % (n=272/293)	0 %	3.3 %[α,#] [illegible]
Leukocytosis	0.3 % (n=1/293)	2.1 % (n=6/293)	2.1 % (n=6/293)	7.2 % (n=21/293)	88.4 % (n=259/293)	4.2 %[β] (n=5/120)	4.2 % ([illegible]
Lymphocytosis	0 %	0.7 % (n=2/293)	0.3 % (n=1/293)	2.7 % (n=8/293)	96.3 % (n=282/293)	1.7 %[β] (n=2/120)	0.8 % ([illegible]
Neutrophilia	0 %	0.3 % (n=1/293)	0.3 % (n=1/293)	2.7 % (n=8/293)	96.6 % (n=283/293)	0.8 % (n=1/120)	2.5 % ([illegible]
Basophilia	0.3 % (n=1/293)	0 %	0.3 % (n=1/293)	2.7 % (n=8/293)	96.6 % (n=283/293)	0.8 % (n=1/120)	3.3 % ([illegible]
Monocytosis	0 %	0.3 % (n=1/293)	0 %	2.1 % (n=6/293)	97.6 % (n=286/293)	2.5 % (n=3/120)	5 % (n=[illegible]
Eosinophilia	0.3 % (n=1/293)	1.0 % (n=3/293)	0.7 % (n=2/293)	2.4 % (n=7/293)	95.2 % (n=279/293)	0.8 % (n=1/120)	4.2 % ([illegible]

Legend: IG s– Immunoglobulins

New insights into immunological involvement in Congenital Disorders of Glycosylation (CDG) from a people-centric approach
Supplementary Material

*A male patient (11-20 yrs) presented improvement of IGs levels after several years of IgG infusions.

#A male patient (4-10 yrs) reported high IgG and low IgA in the last tests.

$A male patient (4-10 yrs)wais described to have had T-lymphopenia (low T CD8+), as well as partial lymphocyte proliferation defect at 18 months. Both parameters have normalized with age.

†Male patient (11-20 yrs) reported to have constant pancytopenia.

αA female patient (11-20 yrs) was reported to have had high IgG1 and IgG4 in the last tests.

βSporadic T lymphopenia (CD3, CD4, CD8); high CD3/DR (absolute counts too), CD16+56+ (NK cells) and CD19 (B cells) counts and increased CD4/8. Low interferon-γ production in response to phytohaemagglutinin (PHA) and high production of lymphotoxin (LT) in response to PHA and concanavalin A (ConA). Low total complement activity, with normal levels of C3 and C4. Low neutrophilic bactericidal activity with normal neutrophil oxidative burst as well as sporadically elevated IgG and IgA levels.

Table S16 – **Information needs related to immunology topics among control, PMM2-CDG and non-PMM2-CDG participants according to immune status.** The numbers and percentages of participants with and without more information requirements is shown for all the groups included in the study. No statistically significant differences were seen among the information needs of participants with and without immunological involvement. However, PMM2-CDG respondents have higher and more consistent information needs than other participants, whilst controls have the lowest information needs.

	**Control group**		**PMM2-CDG group**		**non-PMM2-CDG group**	
**Information needs**	More information on immunology	Do not need more information on immunology	More information on CDG and immunology	Do not need more information on CDG and immunology	More information on CDG and immunology	Do not need more information on CDG and immunology
**Immunological status**	**With immunological involvement**					
**Nº of participants**	**92**/182	**90**/182	**69**/82	**13**/82	**56**/70	**14**/70
**%**	50.55	49.45	84.15	15.85	80	20
**Immunological status**	**Without immunological involvement**					
**Nº of participants**	**79**/159	**80**/159	**22**/27	**5**/27	**11**/16	**5**/16
**%**	49.69	50.31	81.48	18.52	68.75	31.25

**Immunological status**	**Unsolved immune status**					
**Nº of participants**	**5**/7	**2**/7	**11**/13	**2**/13	**2**/2	0
%	71.43	28.57	84.62	15.38	100	

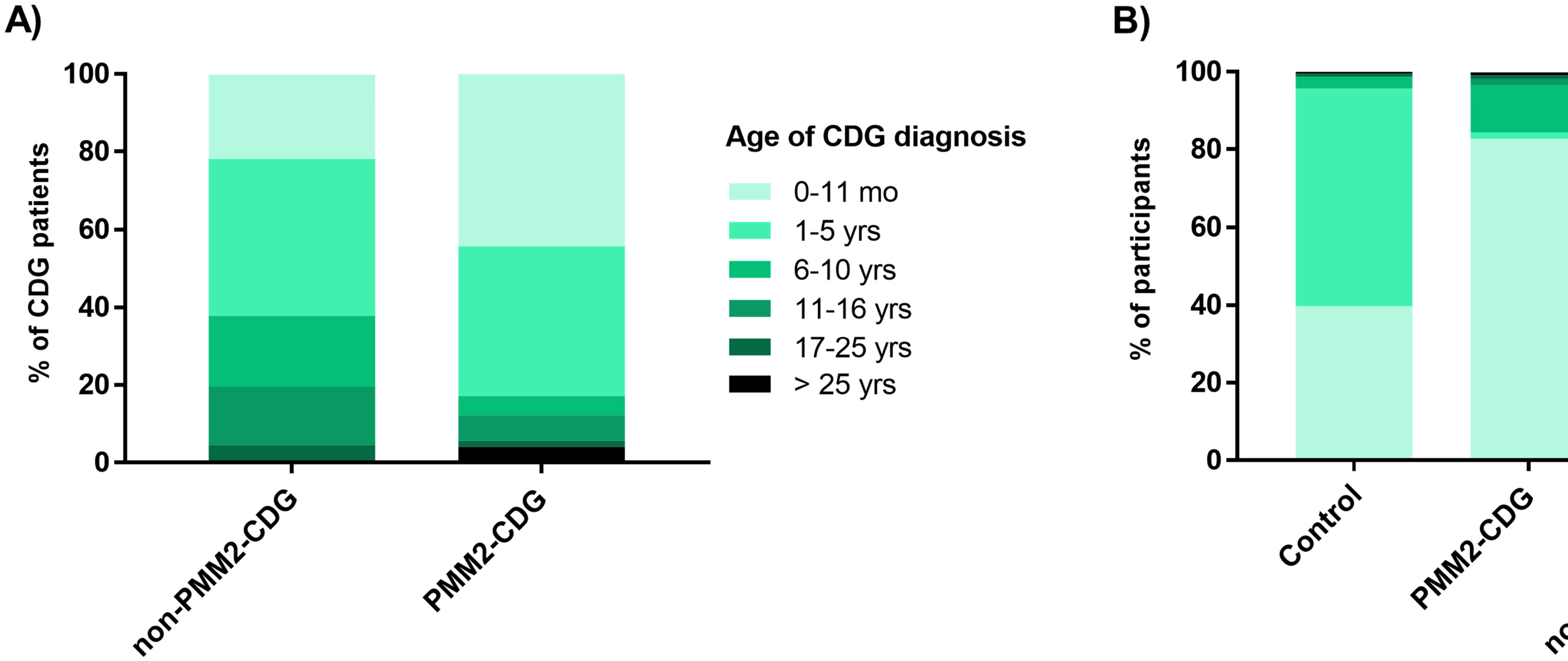
A)
B)
100
80
60
40
20
0
% of CDG patients
% of participants
non-PMM2-CDG
PMM2-CDG
Control
Age of CDG diagnosis
0-11 mo
1-5 yrs
6-10 yrs
11-16 yrs
17-25 yrs
> 25 yrs

Figure S1 – **Characteristics of the ImmunoCDGQ (PMM2-CDG and non-PMM2-CDG groups) and ImmunoHealthyQ (control group) participants.** A) Age of CDG diagnosis of PMM2-CDG and non-PMM2-CDG groups. Most CDG patients were diagnosed before 5 years of age (74.2 %, n=155/209).However, PMM2-CDG patients show a general tendency to get an earlier diagnosis, whereas in non-PMM2-CDG participants 37.9 % (n=33/87) received a diagnosis from 6 years-old onwards. Interestingly, 4.1 % of the PMM2-CDG patients (n=5/122) were diagnosed from 25 years-old onwards. No diagnoses were made after 25 years of age in non-PMM2-CDG; B) Respondents (kinship with the reported participant) of the ImmunoCDGQ and ImmunoHealthyQ. In PMM2-CDG (82.8 %, n=101/122) and non-PMM2-CDG (87.4 %, n=76/87) the wide majority of the respondents were mothers, while in the control group most participants reported themselves (55.9 %, n=195/349).

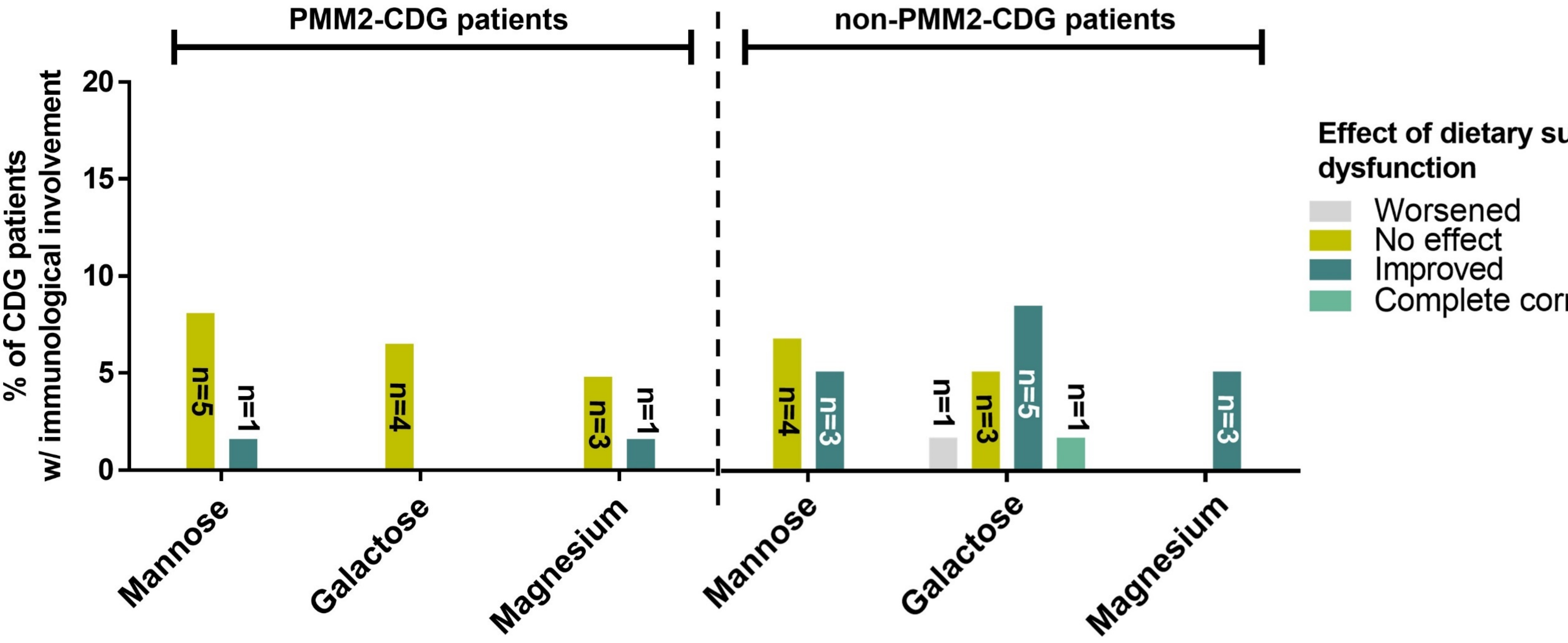
PMM2-CDG patients
non-PMM2-CDG patients
% of CDG patients w/ immunological involvement
20
15
10
5
0
n=5
n=1
n=4
n=3
n=1
n=4
n=3
n=1
n=3
n=5
n=1
n=3
Mannose
Galactose
Magnesium
Mannose
Galactose
Magnesium
Effect of dietary su
dysfunction
Worsened
No effect
Improved
Complete cor

Figure S2 - **Dietary supplementation impact on immunological dysfunction in PMM2-CDG and non-PMM2-CDG patients.** Only 71.6 % (n=62/82) PMM2-CDG patients with immunological involvement answered and 84.3 % (n=59/70) non-PMM2-CDG reporting immunological issues replied to this question. The majority of PMM2-CDG (80.65 %, n=50/62) and non-PMM2-CDG (64.41 %, n=38/59) patients had never or are unaware of ever taking dietary supplements - namely, sugars and trace elements - or of their effect on the immune system/response.

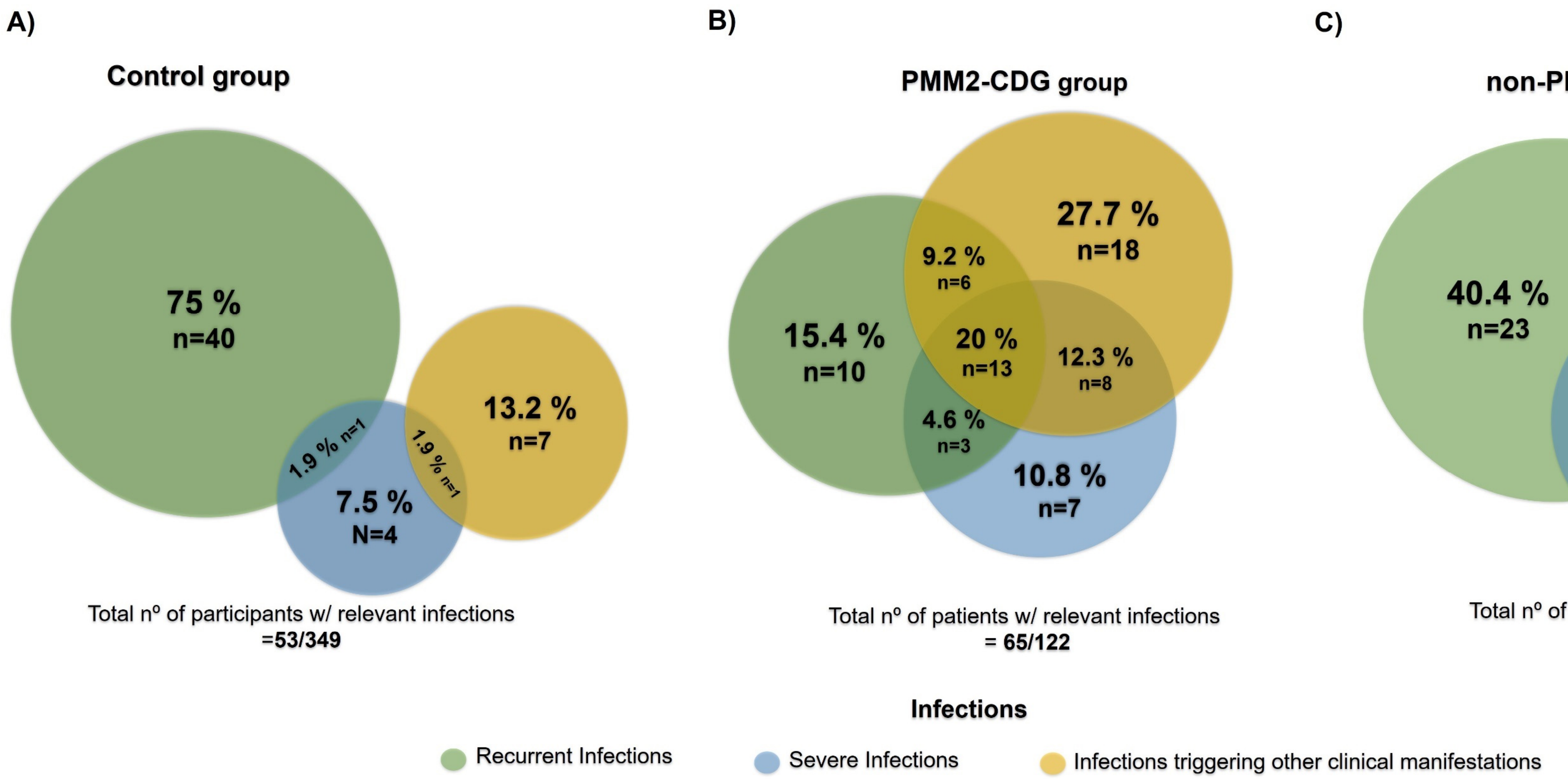
A)
Control group
75 %
n=40
1.9 % n=1
7.5 %
N=4
1.9 % n=1
13.2 %
n=7
Total nº of participants w/ relevant infections
=53/349
B)
PMM2-CDG group
27.7 %
n=18
9.2 %
n=6
15.4 %
n=10
20 %
n=13
12.3 %
n=8
4.6 %
n=3
10.8 %
n=7
Total nº of patients w/ relevant infections
= 65/122
C)
non-Pl
40.4 %
n=23
Total nº of
Infections
Recurrent Infections
Severe Infections
Infections triggering other clinical manifestations

Figure S3 - **Infection profile (frequency and severity classification).** Infection frequency and severity distribution in A) control, B) PMM2-CDG and C) non-PMM2-CDG participants. Most PMM2-CDG patients (53.3 %, n= 65/122) have experienced relevant infections, while only 15.2 % (n=53/349) of the control group reported similar issues. Reports of relevant infection-related issues are significantly higher in PMM2-CDG when compared to the control. Statistical significance refers to the comparison of the total number of PMM2-CDG and control participants with and without infections and was calculated with the Fisher Exact test (p =1.30e^{-15}(****)/ OR=6.34). While control participants mostly described infections are recurrent (75 %, n=40/53), PMM2-CDG participants reported infections to be more clinically complex, with most infections being reported as triggers to other clinical manifestations. The prevalence of relevant infections among non-PMM2-CDG patients is of 65.5% (n=57/87). Most infections observed in these patients were described as recurrent (77.2 %, n=44/57), but in 45.6 % (n=26/57) they were reported to be also severe.

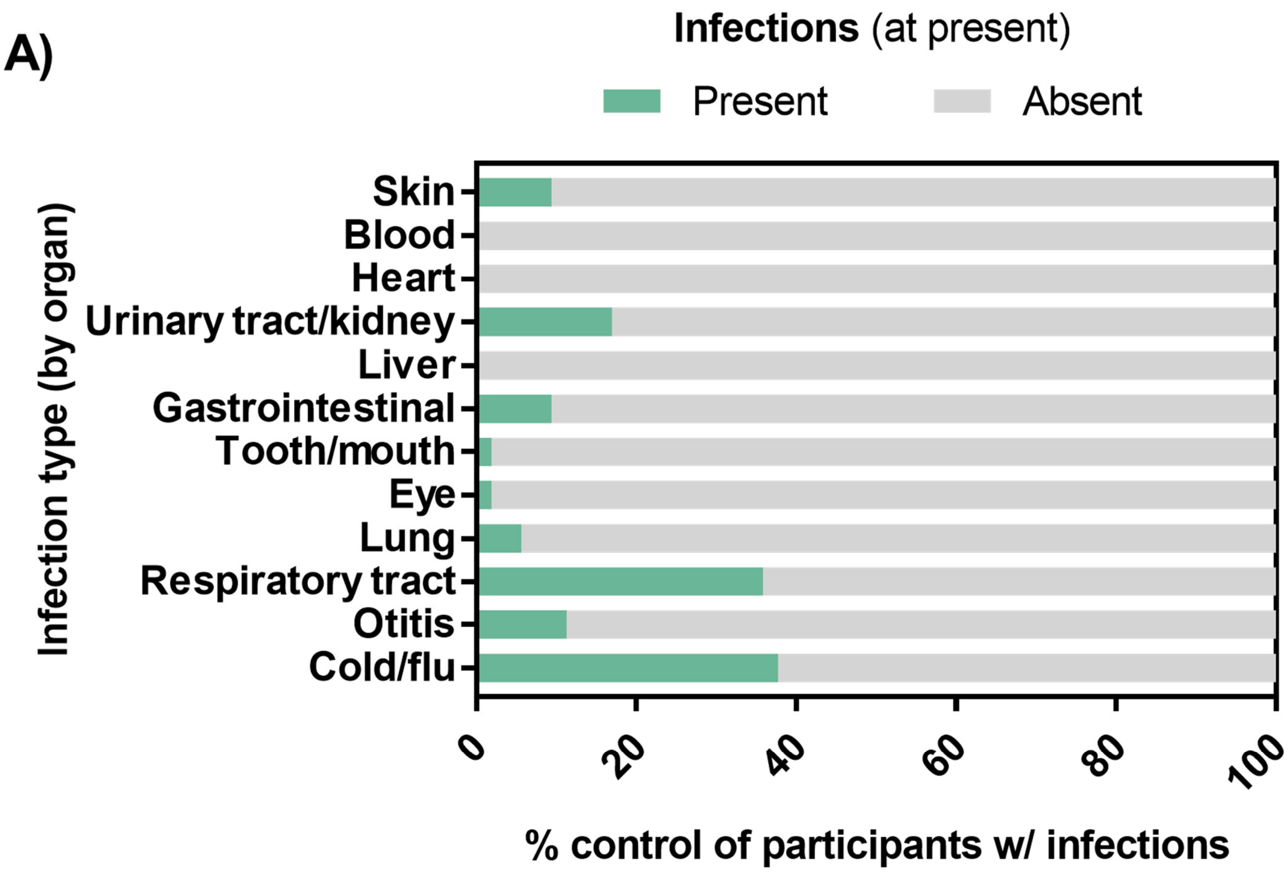
A)
Infections (at present)
Present
Absent
Infection type (by organ)
Skin
Blood
Heart
Urinary tract/kidney
Liver
Gastrointestinal
Tooth/mouth
Eye
Lung
Respiratory tract
Otitis
Cold/flu
0
20
40
60
80
100
% control of participants w/ infections

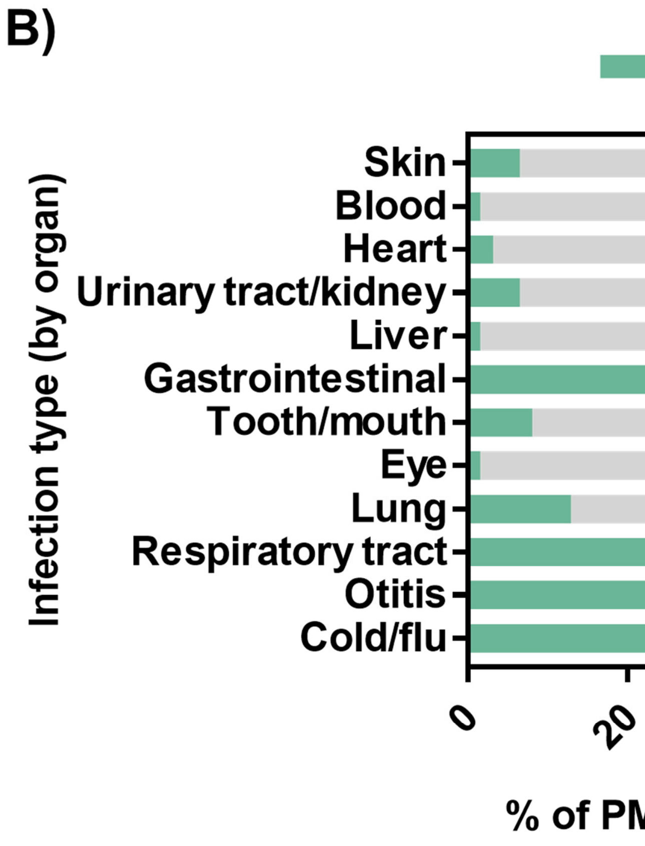
B)
Infection type (by organ)
Skin
Blood
Heart
Urinary tract/kidney
Liver
Gastrointestinal
Tooth/mouth
Eye
Lung
Respiratory tract
Otitis
Cold/flu
0
20

Figure S4 – **Organ-specific infections reported by control and PMM2-CDG at the time of participation in the study.** Prevalence of infection types (by organ) in the A) control sample and B) in PMM2-CDG patients. 72.3 % of PMM2-CDG (n=45/62) and 71.7 % of control participants (n=38/53) reported infections. Collectively, respiratory infections (including cold/flu, respiratory tract and otitis) were the most identified in the control and PMM2-CDG groups. However, gastrointestinal infections were significantly more frequent in PMM2-CDG patients (24.2 %, n=15/62) at the time of participation in the study. Statistical significance – calculated with the Fisher Exact Test - refers to the comparison of participants who reported GI infections against those who did not report them both in PMM2-DG and in the control (p=0.48 and OR= 3.1).

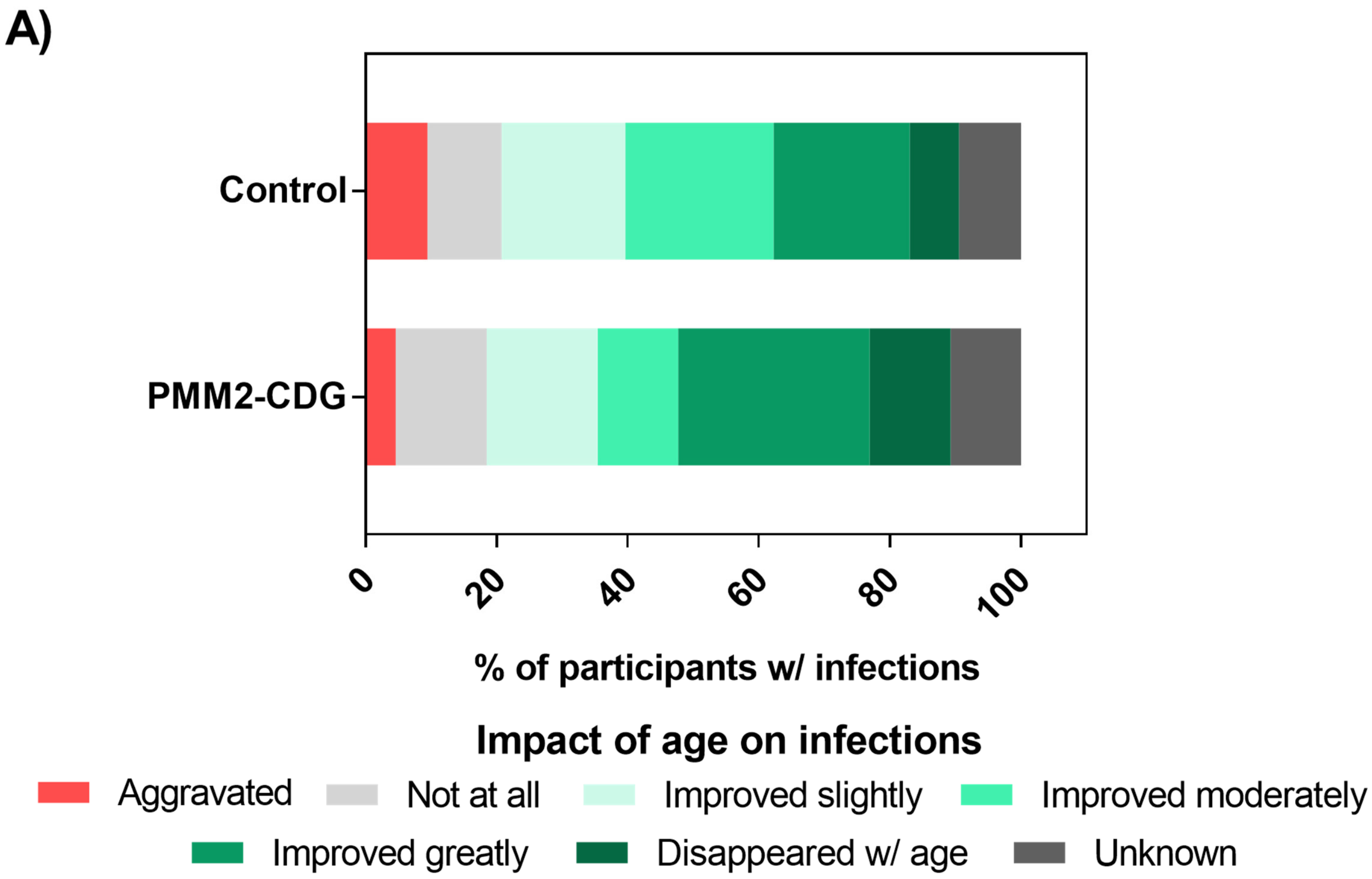
A)
Control
PMM2-CDG
0
20
40
60
80
100
% of participants w/ infections
Impact of age on infections
Aggravated
Not at all
Improved slightly
Improved moderately
Improved greatly
Disappeared w/ age
Unknown

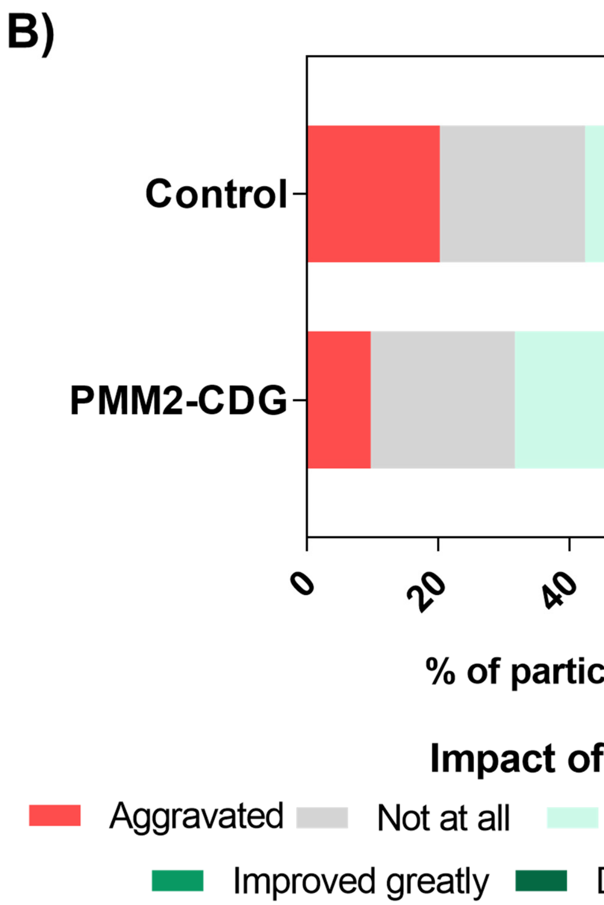
B)
Control
PMM2-CDG
0
20
40
Aggravated
Not at all
Improved greatly

Figure S5 – **Age impact on infection and allergy prevalence and severity.** A) Infection modulation by age; B) Allergy modulation by age. While for infections a tendential positive impact of ageing on infection severity/frequency for allergy that tendency is not as marked, both for PMM2-CDG and controls.

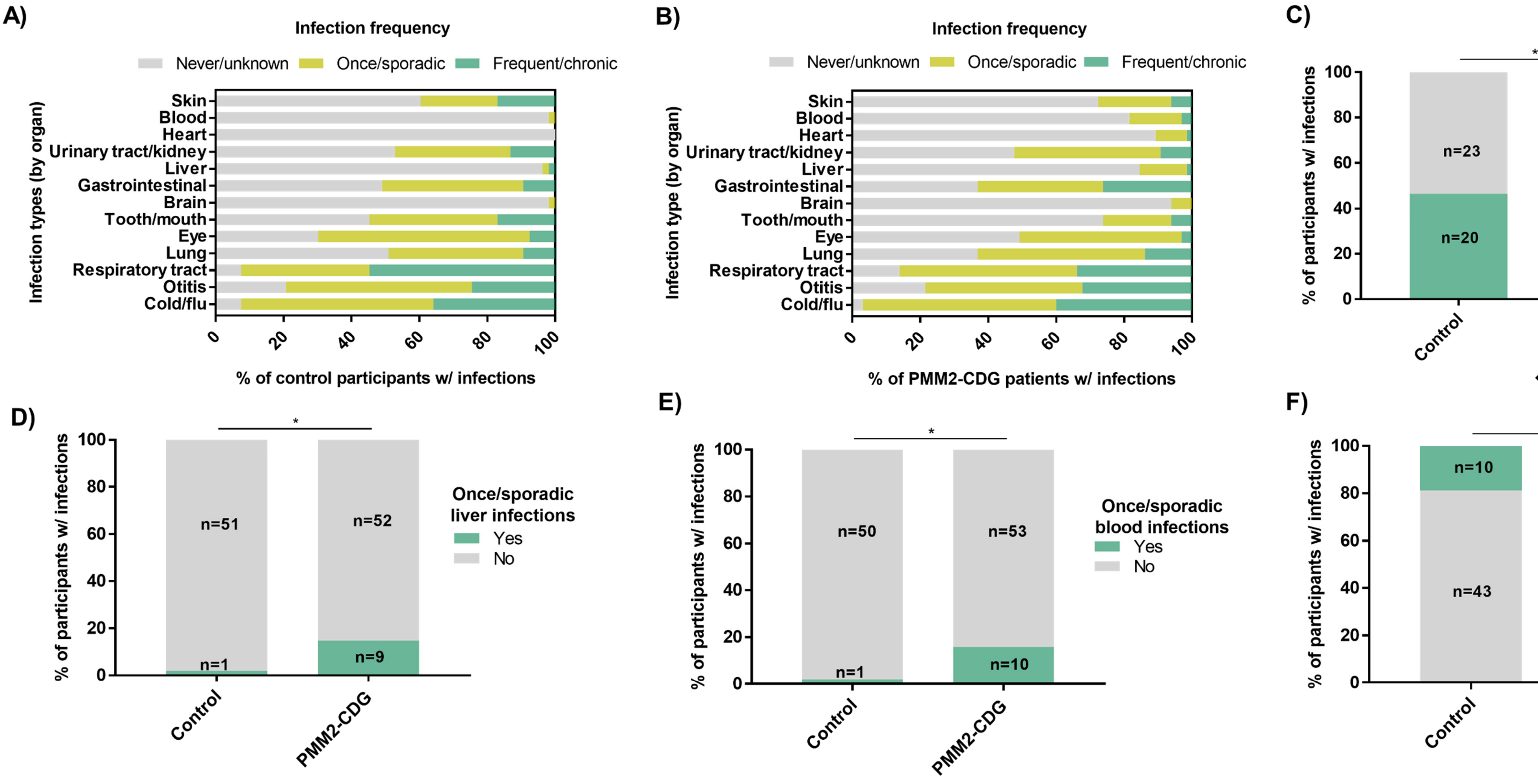
A)
Infection frequency
Never/unknown
Once/sporadic
Frequent/chronic
Infection types (by organ)
Skin
Blood
Heart
Urinary tract/kidney
Liver
Gastrointestinal
Brain
Tooth/mouth
Eye
Lung
Respiratory tract
Otitis
Cold/flu
0
20
40
60
80
100
% of control participants w/ infections
B)
Infection frequency
Never/unknown
Once/sporadic
Frequent/chronic
Infection type (by organ)
Skin
Blood
Heart
Urinary tract/kidney
Liver
Gastrointestinal
Brain
Tooth/mouth
Eye
Lung
Respiratory tract
Otitis
Cold/flu
0
20
40
60
80
100
% of PMM2-CDG patients w/ infections
C)
% of participants w/ infections
100
80
60
40
20
0
n=23
n=20
Control
D)
*
% of participants w/ infections
100
80
60
40
20
0
n=51
n=52
n=1
n=9
Control
PMM2-CDG
Once/sporadic
liver infections
Yes
No
E)
*
% of participants w/ infections
100
80
60
40
20
0
n=50
n=53
n=1
n=10
Control
PMM2-CDG
Once/sporadic
blood infections
Yes
No
F)
% of participants w/ infections
100
80
60
40
20
0
n=10
n=43
Control

Figure S6 – **Infection types (per organ/system) at the time when infections were more frequent/severe and infection-caused permanent damage.** Frequency of infection (by organ) at the time when infections were more frequent/severe A) in control and B) PMM2-CDG participants. Participants answering “One-time” and “sporadic” infections; “frequent” and “chronic” as well as “never happened” and “unknown” were paired. One-time and sporadic C) tooth/mouth (p= 0.0102(*)/OR= 0.32), D) liver (p= 0.020(*)/OR =8.70) and E) blood infections (p= 0.022(*)/OR =9.30) relative prevalence in PMM2-CDG vs control participants. Statistical significance refers to the comparison between participants reporting one-time or sporadic infections (which were grouped) and participants who never experienced these infections. F) Prevalence of reported/known infection-associated permanent damage. PMM2-CDG patients (38.4 %, n=25/65) were reported to have more permanent infection-associated damage than the controls (18.9 %, n=10/53) (p=0.026(*)/OR=2.67). Statistical significance refers to the comparison between participants reporting and not reporting permanent infection-associated damage. Both PMM2-CDG and control participants reported permanent damage to the lungs/airways, liver, speech/communication, and hearing. However, only PMM2-CDG participants reported heart (n=2/25), feeding/oral regression(n=1/25), seizure (n=10/25) and stroke-like (n=11/25) associated damage. A control participant also described permanent bladder damage. Of note, 13.9 % (n=9/65) of PMM2-CDG and 13.2 % of control participants (n=7/53) were uncertain if any infection-related lasting damage had occurred. This is probably related to the fact that infection-caused permanent damage can be hard to confirm or rule out. All statistical significance calculated with Fisher Exact Test.

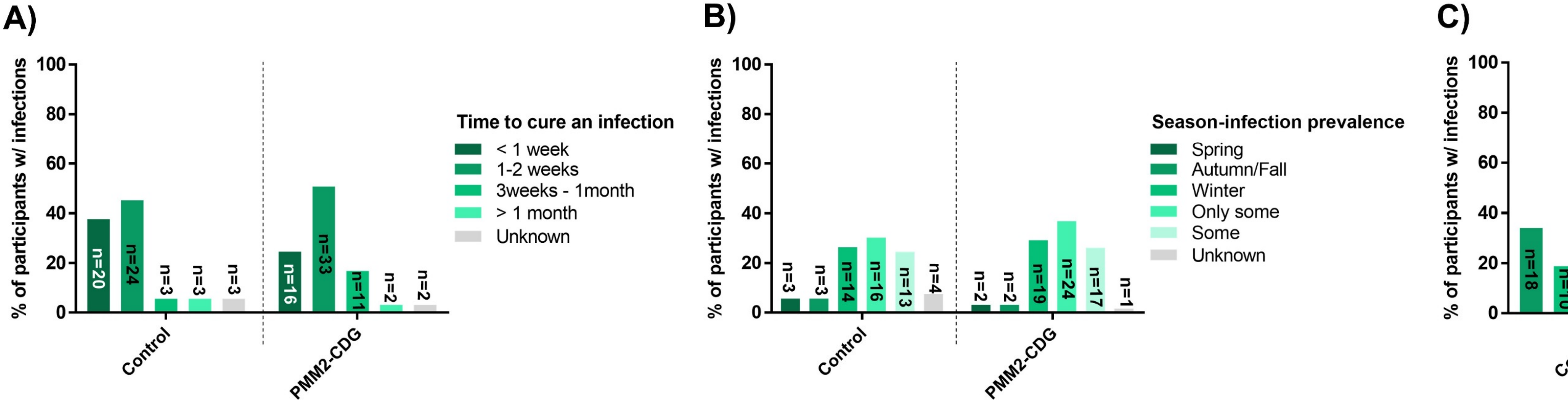

Figure S7 – **Time to cure infections, their association with seasons and causing infectious agents.** A) Time to cure an infection. Time that infections usually take to completely resolve. No statistical differences were found but PMM2-CDG patients (73.8 %, n=48/65 took ≥ 1-2 weeks) showed a tendency to take a longer time to get rid of infections (OR= 1.93). B) Prevalence of infections by season. No season-dependent or independent relationship between infections was found in either group. However, neither control nor PMM2-CDG participants identified summer as the season when more infections happened. C) Infectious agents. No specific type of infectious agent was predominantly named. Nevertheless, bacteria were the most identified infectious agent in controls (33.9 %, n=18/53), while in PMM2-CDG a more equal identification of infectious agent was made. PMM2-CDG participants (27.7 %, n=18/65) identified virus more frequently than controls (18.9 %, n=10/53).

New insights into immunological involvement in Congenital Disorders of Glycosylation (CDG) from a people-centric approach

Supplementary Material

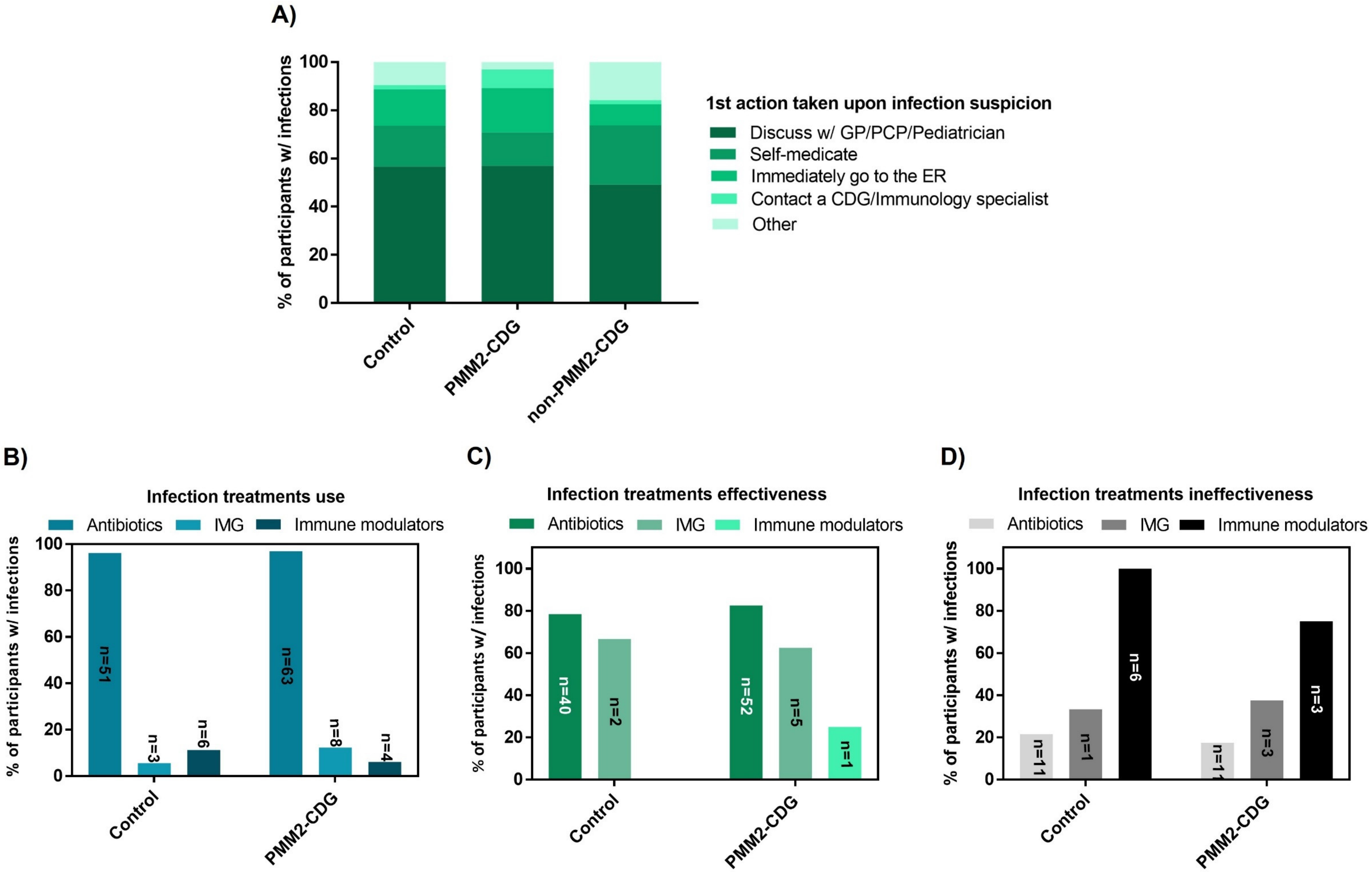

New insights into immunological involvement in Congenital Disorders of Glycosylation (CDG) from a people-centric approach

Supplementary Material

Figure S8 – **Infection treatments and actions taken upon infection suspicion**. A) First action taken when infection is suspected. B) Antibiotics, intravenous immunoglobulins and immune modulators use, C) effectiveness and D) ineffectiveness prevalence among PMM2-CDG and control groups with relevant infections. No participants reported stem cell transplant as an infection treatment Legend: ER – Emergency room; IVIG – intravenous immunoglobulins; PCP – Principal care physician.

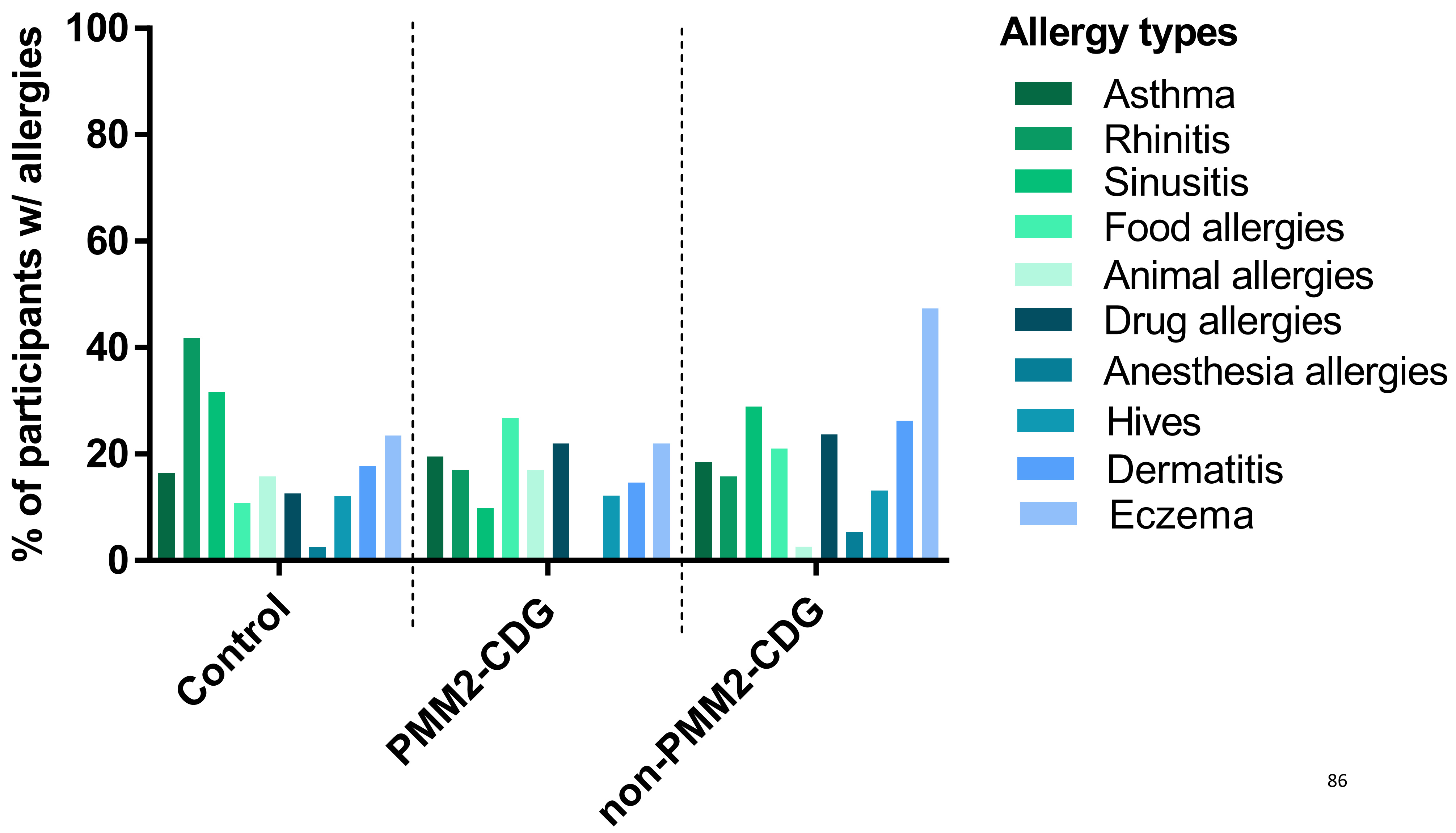
% of participants w/ allergies
100
80
60
40
20
0
Control
PMM2-CDG
non-PMM2-CDG
Allergy types
Asthma
Rhinitis
Sinusitis
Food allergies
Animal allergies
Drug allergies
Anesthesia allergies
Hives
Dermatitis
Eczema

Figure S9 - **Allergy prevalence.** A) Prevalence of specific allergies in the control, PMM2-CDG and non-PMM2-CDG participants. Overall allergy presence was lower in the PMM2-CDG (33%, n=41/122) and non-PMM2-CDG groups (43.7 %, n=38/87) when compared to control group (45 %, n=158/349). In PMM2-CDG specific allergy distribution is quite more balanced than those observed for the control and non-PMM2-CDG groups. In controls there is a higher prevalence of respiratory allergies and in non-PMM2-CDG a higher incidence of skin allergies. Among PMM2-CDG only food allergies were significantly more elevated when compared to the control (p=0.020 (*)/OR=3.02). These quantitative results are in accordance with comments made by PMM2-CDG respondents that in two cases mentioned egg allergies, while another also described positive skin tests results for wheat, walnut, cashew and sesame. Statistical significance – determined using the Fisher Exact test - refers to the comparison between participants reporting food allergies and those who did not both in PMM2-CDG and controls.

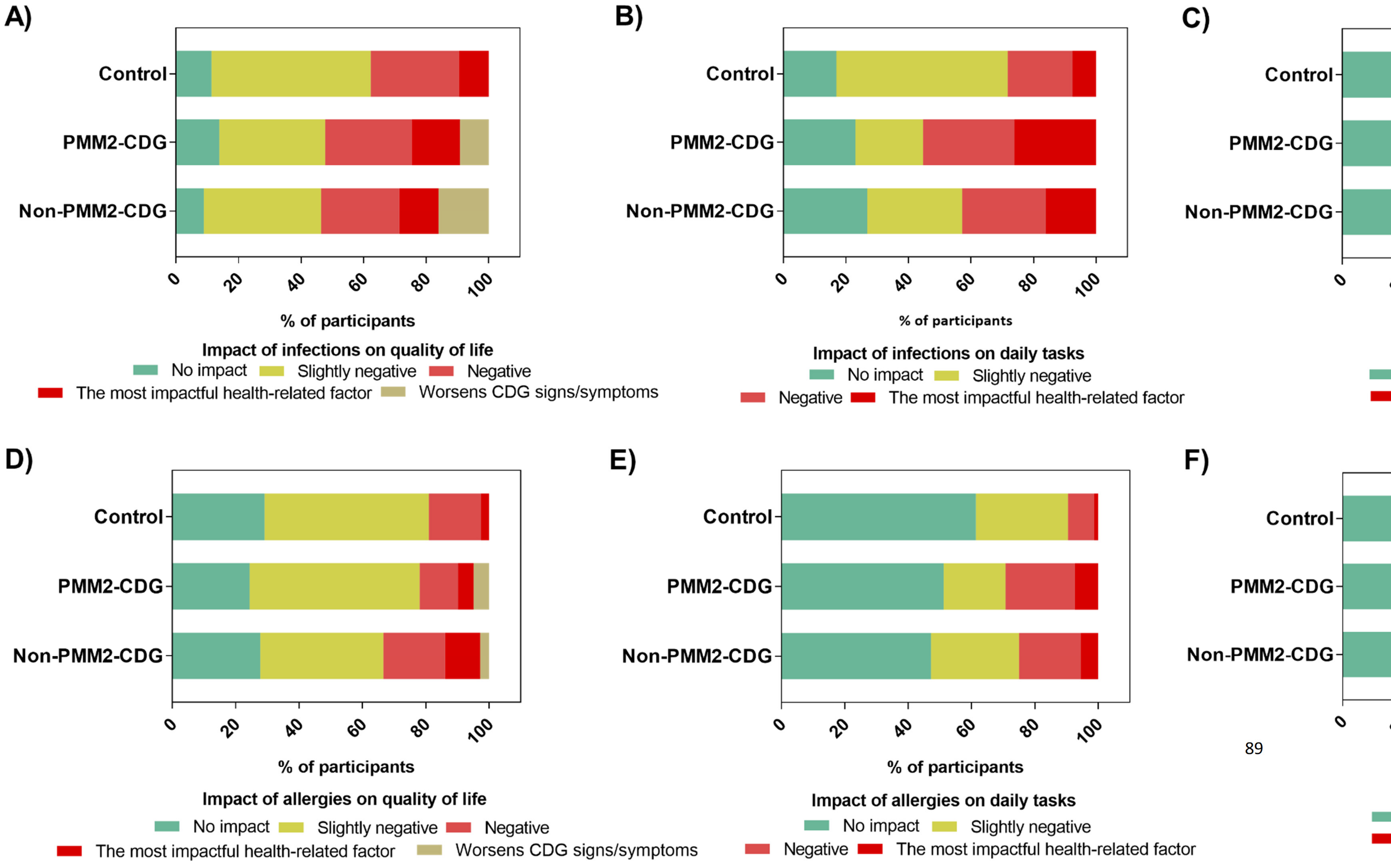
A)
Control
PMM2-CDG
Non-PMM2-CDG
0
20
40
60
80
100
% of participants
Impact of infections on quality of life
No impact
Slightly negative
Negative
The most impactful health-related factor
Worsens CDG signs/symptoms
B)
Control
PMM2-CDG
Non-PMM2-CDG
0
20
40
60
80
100
% of participants
Impact of infections on daily tasks
No impact
Slightly negative
Negative
The most impactful health-related factor
C)
Control
PMM2-CDG
Non-PMM2-CDG
0
D)
Control
PMM2-CDG
Non-PMM2-CDG
0
20
40
60
80
100
% of participants
Impact of allergies on quality of life
No impact
Slightly negative
Negative
The most impactful health-related factor
Worsens CDG signs/symptoms
E)
Control
PMM2-CDG
Non-PMM2-CDG
0
20
40
60
80
100
% of participants
Impact of allergies on daily tasks
No impact
Slightly negative
Negative
The most impactful health-related factor
F)
Control
PMM2-CDG
Non-PMM2-CDG
0

New insights into immunological involvement in Congenital Disorders of Glycosylation (CDG) from a people-centric approach

Supplementary Material

Figure S10 – **Infection and allergy impact on quality of life (QoL) and perceived severity in control, PMM2-CDG and non-PMM2-CDG groups.** A) Impact of infections on general QoL; B) Impact of infections on everyday tasks; C) Perceived infection severity. PMM2-CDG patients had significantly higher perceived infection severity. Statistical significance – calculated with Fisher Exact Test - refers to the comparison between participants rating infections as mild and those who described infections as moderate, severe or very severe (which were grouped in one single variable) (p= 0.019(*)/OR=2.64). Participants who were unaware or uncertain of infection severity were excluded from this analysis; D) Impact of allergies on general QoL; E) Impact of allergies on everyday tasks; F) Perceived allergy severity.

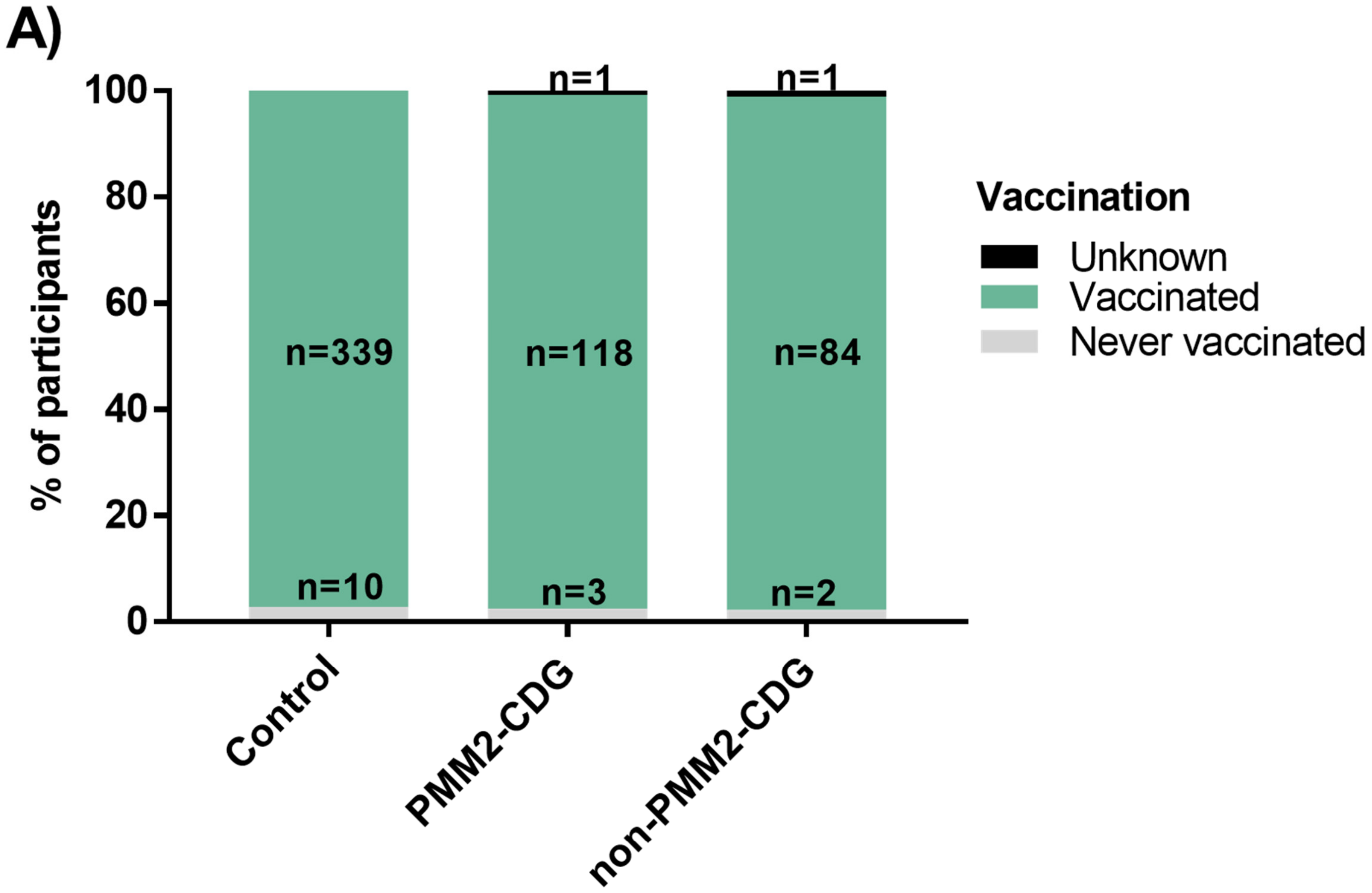
A)
100
80
60
40
20
0
% of participants
n=1
n=1
n=339
n=118
n=84
n=10
n=3
n=2
Control
PMM2-CDG
non-PMM2-CDG
Vaccination
Unknown
Vaccinated
Never vaccinated

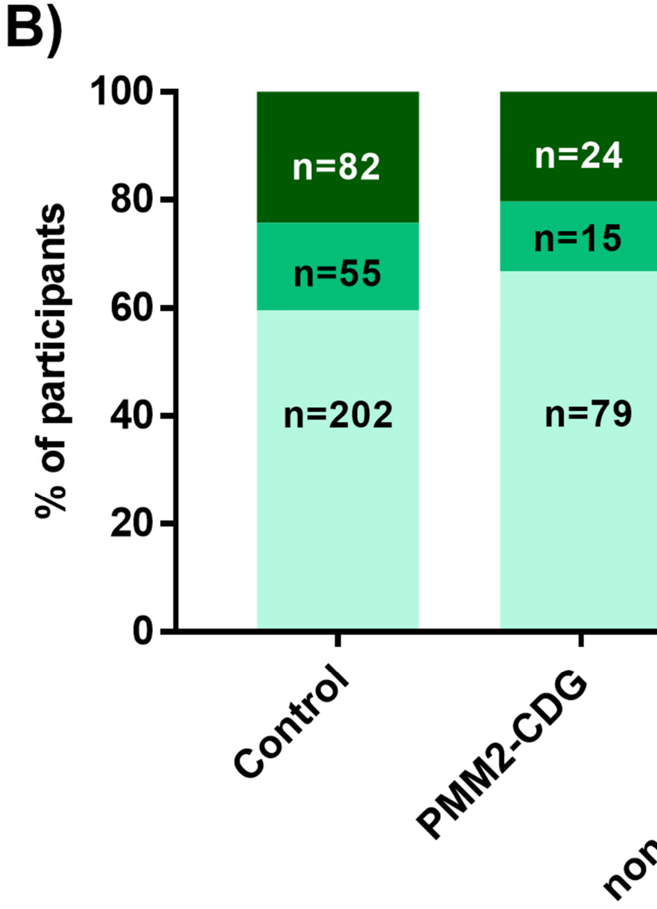
B)
100
80
60
40
20
0
% of participants
n=82
n=24
n=55
n=15
n=202
n=79
Control
PMM2-CDG

Figure S11 - **Vaccination adherence and monitoring**. A) Vaccination adherence. In this case, being vaccinated implies that the participant had received at least one vaccine. Almost all participants reported being vaccinated in control (97.1%, n=339/349), PMM2-CDG (96.7%, n=118/122) and non-PMM2-CDG (96.6 %, n=84/87); B) Reported vaccination monitoring by measurement of antibody titers. In the variable “Monitored” participants reporting having had vaccination monitored (by antibody titers determination) “Once”, “Constantly” and “In every vaccination” were combined. Only small subset of participants in PMM2-CDG (12.7 %, n=15/118), non-PMM2-CDG (15.5 %, n=13/84) and control (16.2 %, n=55/339) participants reported ever having monitored the vaccination response.

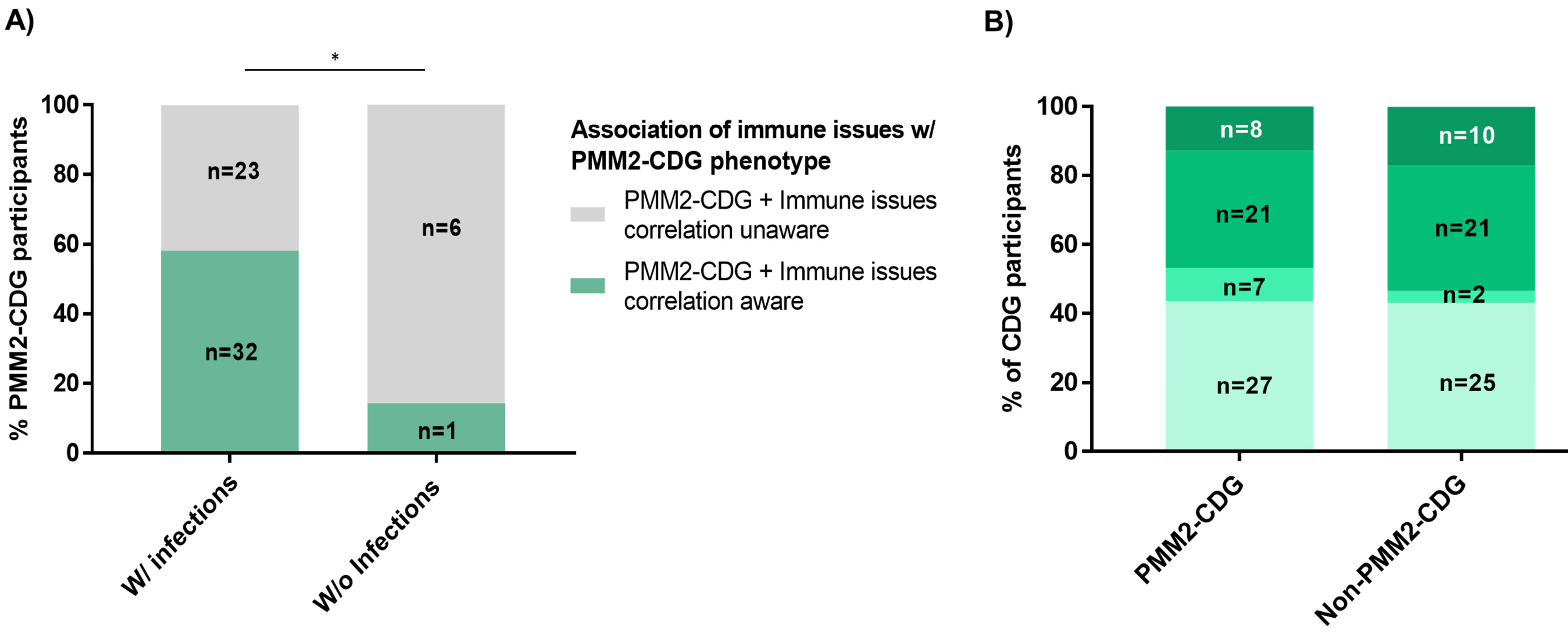
A)
*
100
80
60
40
20
0
% PMM2-CDG participants
n=23
n=32
n=6
n=1
W/ infections
W/o Infections
Association of immune issues w/ PMM2-CDG phenotype
PMM2-CDG + Immune issues correlation unaware
PMM2-CDG + Immune issues correlation aware
B)
100
80
60
40
20
0
% of CDG participants
n=8
n=21
n=7
n=27
n=10
n=21
n=2
n=25
PMM2-CDG
Non-PMM2-CDG

Figure S12 – **Immune awareness**. A) Correlation between immune issues and PMM2-CDG. PMM2-CDG patients with relevant infections were more likely to have had their immune-related manifestations related to their underlying CDG (49.2%, n=32/65). Statistical significance refers to the comparison between participants reporting having a clinician who associated immune issues with CDG and who were followed by an immunologist/rheumatologist were grouped ("PMM2-CDG + Immune issues correlation aware") and participants claiming immune issues had not been associated with CDG by any clinician ("PMM2-CDG + Immune issues correlation unaware") (*p*=0.044 (*)/OR=8.09). B) Association and/or recognition of immune-related manifestations as part of PMM2-CDG and non-PMM2-CDG (response rate of 76.9% in PMM2-CDG – n=63/82 - and 82.9% in non-PMM2-CDG patients, n=58/70). Statistical significance was calculated with Fisher Exact Test.

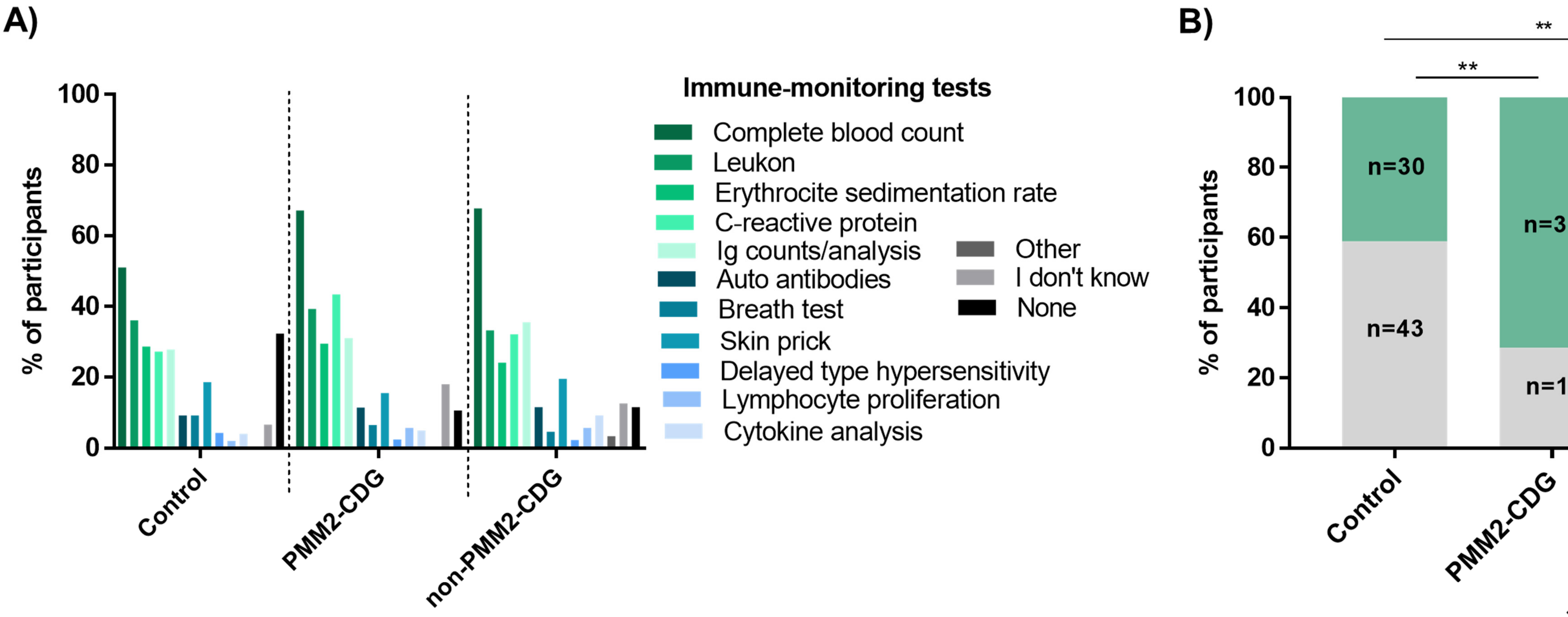
A)
Immune-monitoring tests
Complete blood count
Leukon
Erythrocite sedimentation rate
C-reactive protein
Ig counts/analysis
Auto antibodies
Breath test
Skin prick
Delayed type hypersensitivity
Lymphocyte proliferation
Cytokine analysis
Other
I don't know
None
% of participants
100
80
60
40
20
0
Control
PMM2-CDG
non-PMM2-CDG
B)
**
**
n=30
n=43
% of participants
Control
PMM2-CDG

Figure S13 - **Immune testing**. A) Types of immune tests reported by control, PMM2-CDG and non-PMM2-CDG participants. Most participants recognised having done a complete blood count (CBC); B) Immune testing frequency. PMM2-CDG (*p*=0.0015(**)/OR=3.54) and non-PMM2-CDG (*p*=0.0022(**)/OR=3.82) patients have their immune parameters tested more frequently than controls. Statistical significance refers to the comparison between participants reporting to have done immune tests once and others reporting having done the tests with greater periodicity (at least, twice) which were combined into the "> Once" group. Participants unaware or uncertain of the frequency of testing were excluded from this analysis. Only participants reporting the presence of immune-related manifestations (prior to this question) and able of communicating the frequency of testing had to reply to this question. Statistical significance was calculated with Fisher Exact Test.

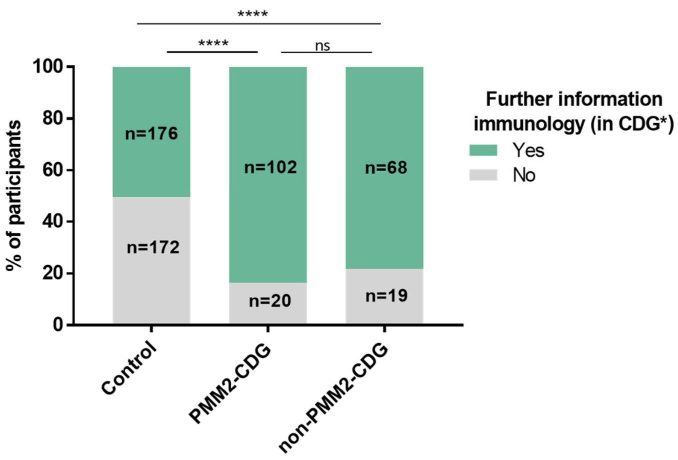
****
****
ns
100
80
60
40
20
0
% of participants
n=176
n=172
n=102
n=20
n=68
n=19
Control
PMM2-CDG
non-PMM2-CDG
Further information immunology (in CDG*)
Yes
No

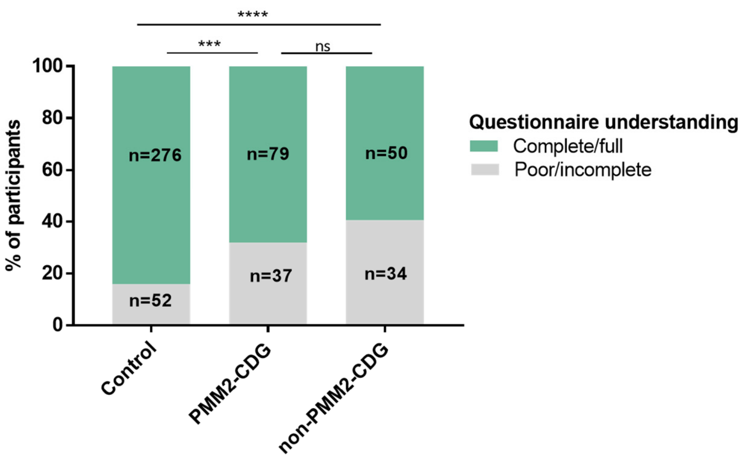
****
***
ns
100
80
60
40
20
0
% of participants
n=276
n=52
n=79
n=37
n=50
n=34
Control
PMM2-CDG
non-PMM2-CDG
Questionnaire understanding
Complete/full
Poor/incomplete

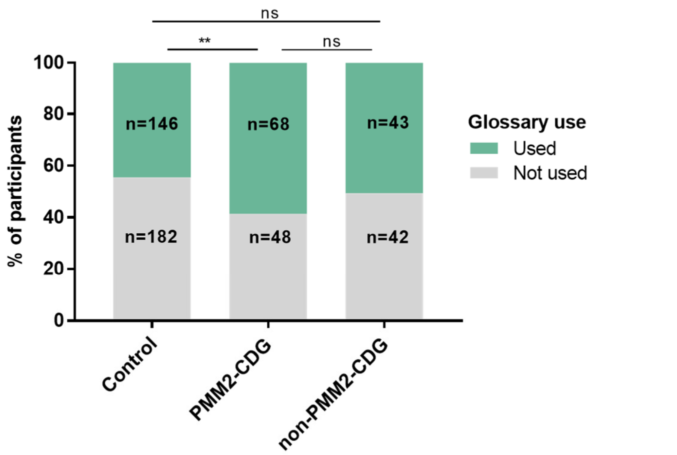
ns
**
ns
100
80
60
40
20
0
% of participants
n=146
n=182
n=68
n=48
n=43
n=42
Control
PMM2-CDG
non-PMM2-CDG
Glossary use
Used
Not used

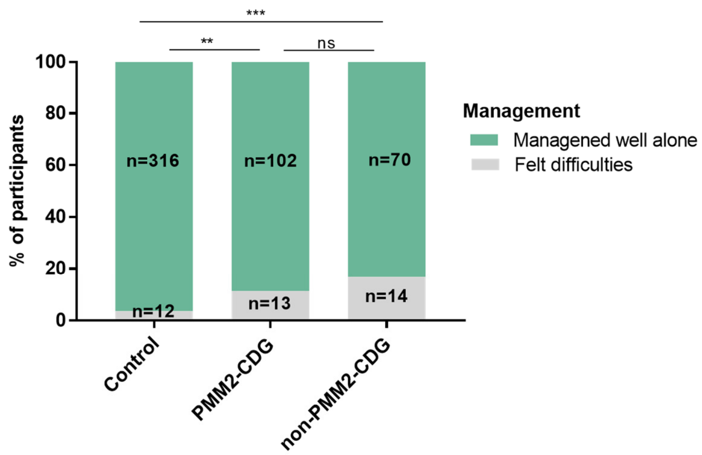
***
**
ns
100
80
60
40
20
0
% of participants
n=316
n=12
n=102
n=13
n=70
n=14
Control
PMM2-CDG
non-PMM2-CDG
Management
Managened well alone
Felt difficulties

Figure S14 – **Immunology-related information needs, glossary use, questionnaire understandability and management.** A) Immunology-related information needs. Most participants would like to have access to more information on immunology topics. PMM2-CDG and non-PMM2-CDG participants were asked if they would like to receive more information on CDG and immunology, while control participants were asked if they would like to get more information on immunology. Immunology-related information needs were more significant among PMM2-CDG (p-value=6.055e^{-11}(****)/OR=4.97) and non-PMM2-CDG patients (*p*=2.023e^{-06}(***)/OR=3.54). Statistical significance refers to the comparison between participants responding yes and no to requesting more information; B) Questionnaires understandability. Most participants reported a complete understanding of the questionnaire, but control participants reported a significantly better understanding than PMM2-CDG (*p*=0.00040(***)/OR=0.40) and non-PMM2-CDG participants (*p*=1.903e^{-06}(***)/OR=0.27). Statistical significance refers to the comparison between participants answering "Yes, I understood what was being asked in every question" (constituted the "Complete/full understanding" group)

and were participants choosing any of the other answer options which were combined into the "Poor/incomplete understanding" group. Response rate = 94 % of control (n=328/349), 95.1% of PMM2-CDG (n=116/122) and 96.6% of non-PMM2-CDG participants (n=84/87); C) Glossary use while completing the questionnaires. PMM2-CDG participants (58.6%, n=68/116) reported resorting to glossaries significantly more than other participants (p=0.0096 (**)/OR=1.76). Statistical significance refers to the comparison between participants replying "I did not need to refer to the glossary" (formed the "Not used" variable) and all the other participants who were grouped into the "Used" variable. Response rate = 93.9% of control (n=328/349), 95.1% of PMM2-CDG (n=116/122) and 97.7% of non-PMM2-CDG participants (n=85/87); D) Management of the experience of study participants. Most participants felt they managed well alone. Controls also exhibited a better management experience than PMM2-CDG (p=0.0042(**)/OR=0.30) and non-PMM2-CDG participants (p=0.00011(***)/OR=0.19). Statistical significance refers to the comparison between participants replying "No, I could manage well on my own" (represented by the variable "Managed well alone") and participants who chose other answer options making up the "Felt difficulties" group. Response rate = 93.9% of control (n=328/349), 94.3% of PMM2-CDG (n=115/122) and 96.6% of non-PMM2-CDG participants (n=84/87). All statistical significance was determined by the Fisher exact test.

Figure S15 – **Overall analysis of respondents' views about the questionnaires' understandability, glossary use/usefulness, and their participation experience/management.** A) Control; B) PMM2-CDG and C) non-PMM2-CDG groups are represented.

A)

Understandability

Glossary usefulness

	Glossary usefulness	*Experience/ Management*	*Glossary usefulness*	*Experience/ Management*	*Glossary usefulness*	*Experience/ Management*
Overall understandability	**Yes, I understood what was being asked in every question**		**I didn't always understand what was being asked**		**I didn't understand what was being asked for majority of the questions**	
Nº of respondents	**276**		**46**		**6**	
**Glossary/ experience**	**Glossary**	**Experience**	**Glossary**	**Experience**	**Glossary**	**Experience**
	**168** respondents didn't refer to the glossaries	**268** respondents felt they managed well on their own	**11** respondents didn't refer to the glossaries	**40** respondents felt they managed well on their own	**1** respondent didn't refer to the glossaries	**5** respondents felt t managed well on th own
	**94** respondents said the glossaries were very helpful	**4** respondents only asked for help in a few questions	**18** respondents said the glossaries were very helpful	**3** respondents only asked for help in a few questions	**3** respondents said the glossaries were very helpful	
	**11** respondents found the glossaries were only partially helpful	**1** respondent felt out of their depth	**16** respondents found the glossaries were only partially helpful	**3** respondents felt out of their depth	**1** respondent found the glossaries were only partially helpful	**1** respondent felt ou their depth
	**1** respondent said the glossaries weren't helpful at all		**1** respondent said the glossaries weren't helpful at all		**1** respondent said the glossaries weren't helpful at all	

New insights into immunological involvement in Congenital Disorders of Glycosylation (CDG) from a people-centric approach

Supplementary Material

B)

Understandability (arrow pointing left)

Glossary usefulness (arrow pointing up)

	Glossary usefulness	*Experience/ Management*	*Glossary usefulness*	*Experience/ Management*	*Glossary usefulness*	*Experience/ Management*
Overall understandability	Yes, I understood what was being asked in every question		I didn't always understand what was being asked		I didn't understand what was being asked for [illegible] majority of the questions	
Nº of respondents	79		34		3	
Glossary/ experience	Glossary	Experience	Glossary	Experience	Glossary	Experience
	**37** respondents didn't refer to the glossaries	**68** respondents felt they managed well on their own	**9** respondents didn't refer to the glossaries	**31** respondents felt they managed well on their own	**2** respondents didn't refer to the glossaries	**2** respondents felt t[illegible] managed well on th[illegible] own
	**33** respondents said the glossaries were very helpful	**5** respondents only asked for help in a few questions	**10** respondents said the glossaries were very helpful	**1** respondent only asked for help in a few questions	**1** respondent the glossaries were very helpful	**1** respondent only asked for help in a fe[illegible] questions
	**9** respondents found the glossaries were only partially helpful	**4** respondents felt out of their depth	**14** respondents found the glossaries were only partially helpful	**2** respondents felt out of their depth		

C)

Understandability

Glossary usefulness

	Glossary usefulness	*Experience/ Management*	*Glossary usefulness*	*Experience/ Management*	*Glossary usefulness*	*Experience/ Management*	[illegible]
Overall understandability	**Yes, I understood what was being asked in every question**		**I didn't always understand what was being asked**		**I didn't understand what was being asked for the majority of the questions**		
Nº of respondents	**50**		**29**		**6**		
**Glossary/ experience**	**Glossary**	**Experience**	**Glossary**	**Experience**	**Glossary**	**Experience**	
	**31** respondents didn't refer to the glossaries	**45** respondents felt they managed well on their own	**8** respondents didn't refer to the glossaries	**25** respondents felt they managed well on their own	**2** respondents didn't refer to the glossaries	**1** respondents felt they managed well on their own	**41** [illegible]
	16 respondents said the glossaries were very helpful		**12** respondents said the glossaries were very helpful	**2** respondents only asked for help in a few questions			**2** [illegible]
	**3** respondents found the glossaries were only partially helpful	**5** respondents felt out of their depth	**7** respondents found the glossaries were only partially helpful	**1** respondents felt out of their depth	**2** respondents found the glossaries were only partially helpful	**5** respondents felt out of their depth	**12** [illegible]
			**1** respondent said the glossaries weren't helpful at all				[illegible]

New insights into immunological involvement in Congenital Disorders of Glycosylation (CDG) from a people-centric approach

Supplementary Material
